# The PACAP Paradox: Dynamic and Surprisingly Pleiotropic Actions in the Central Regulation of Energy Homeostasis

**DOI:** 10.3389/fendo.2022.877647

**Published:** 2022-06-01

**Authors:** Nikki Le, Sarah Sayers, Veronica Mata-Pacheco, Edward J. Wagner

**Affiliations:** ^1^ Graduate College of Biomedical Sciences, Western University of Health Sciences, Pomona, CA, United States; ^2^ College of Osteopathic Medicine of the Pacific, Western University of Health Sciences, Pomona, CA, United States

**Keywords:** proopiomelanocortin (POMC), pituitary adenylate cyclase activating peptide (PACAP), food addiction, sex difference, A10 dopamine neurons, feeding, homeostatic feeding

## Abstract

Pituitary Adenylate Cyclase-Activating Polypeptide (PACAP), a pleiotropic neuropeptide, is widely distributed throughout the body. The abundance of PACAP expression in the central and peripheral nervous systems, and years of accompanying experimental evidence, indicates that PACAP plays crucial roles in diverse biological processes ranging from autonomic regulation to neuroprotection. In addition, PACAP is also abundantly expressed in the hypothalamic areas like the ventromedial and arcuate nuclei (VMN and ARC, respectively), as well as other brain regions such as the nucleus accumbens (NAc), bed nucleus of stria terminalis (BNST), and ventral tegmental area (VTA) – suggesting that PACAP is capable of regulating energy homeostasis *via* both the homeostatic and hedonic energy balance circuitries. The evidence gathered over the years has increased our appreciation for its function in controlling energy balance. Therefore, this review aims to further probe how the pleiotropic actions of PACAP in regulating energy homeostasis is influenced by sex and dynamic changes in energy status. We start with a general overview of energy homeostasis, and then introduce the integral components of the homeostatic and hedonic energy balance circuitries. Next, we discuss sex differences inherent to the regulation of energy homeostasis *via* these two circuitries, as well as the activational effects of sex steroid hormones that bring about these intrinsic disparities between males and females. Finally, we explore the multifaceted role of PACAP in regulating homeostatic and hedonic feeding through its actions in regions like the NAc, BNST, and in particular the ARC, VMN and VTA that occur in sex- and energy status-dependent ways.

## Introduction

The brain receives and processes metabolic signals from multiple sources such as gut hormones, nutrients, and circulating leptin and insulin levels to modulate energy intake and energy expenditure by sending the information to the brainstem and the hypothalamus (1). The hypothalamus and the brainstem coordinately modulate energy homeostasis by controlling food intake and body weight, autonomic regulation of gastrointestinal function (2), as well as energy expenditure through changes in thermogenesis and locomotor activity (3).The dorsal vagal complex is an important center within the brainstem that process signals received through vagal afferents and projects to the hypothalamus and other regions (4). The dorsal vagal complex consists of the nucleus tractus solitarius (NTS), the dorsal motor nucleus of the vagus, and the area postrema, which is easily accessible to peripheral signals due to the incomplete blood brain barrier surrounding it (5). Vagal afferent fibers emanating from the gut have cell bodies in the nodose ganglion and release glutamate from terminals in the NTS to activate group II metabotropic glutamate receptors in these cells. These second order NTS neurons, in turn, employ an array of different neurotransmitters to control gastric functions (2). The NTS also contain other neuropeptides like proopiomelanocortin (POMC), cocaine- and amphetamine-regulated transcript (CART), and glucagon-like peptide-1 (GLP-1) (5–7), which has been touted as anorexigenic (i.e., appetite suppressing) peptides known for their predictive biomarkers of satiety. Indeed, these neurons are stimulated upon food consumption, in part, through the activation of signal transducer and activator of transcription 3 in response to exogenous leptin (8). A recent paper reported that NTS catecholaminergic and neuropeptide Y (NPY) neurons also play a role in the vagal-mediated gut-brain pathway to both stimulate orexigenesis (i.e., hunger or appetite) and suppress feeding in an opposing manner such that the activation of NTS epinephrine neurons co-expressing NPY triggers food consumption while activation of NTS norepinephrine neurons induces satiety, respectively (9). Vagus nerve transmission from the gut to the NTS is extremely important in sensing distension of the gut wall as well as luminal contents, and either acute or chronic stimulation of vagus nerve causes weight loss and food reduction in rats (2, 10). Gut-derived hormones such as GLP-1, cholecystokinin, and peptide YY are released upon sensing food consumption and upon binding to their receptors (11–14), and the vagus nerve conveys this information to the NTS, which, in turn, relays it to the hypothalamus to induce satiety (7, 15). Beside sending signals to the hypothalamus, the NTS receives descending neuronal projections from both the lateral hypothalamus (LH) and the paraventricular nucleus (PVN) (16, 17). As an example, orexin neurons are highly expressed in the LH, extensively projected to many different brain regions, and the administration of orexin A into the caudal brainstem (18) or the hindbrain (19) causes an increase in food intake.

**Figure 1:**
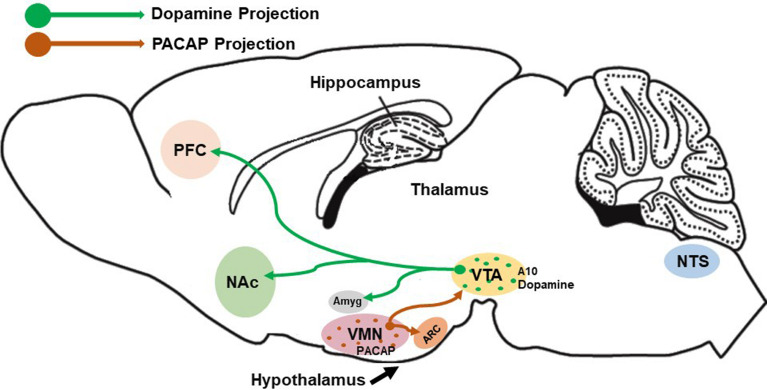
Schematic depiction of VMN PACAP neurons innervating other hypothalamic and mesencephalic structures (e.g., the ARC and VTA, respectively) to regulate homeostatic and hedonic feeding. Postsynaptic targets of these VMN PACAP neurons include POMC neurons and A_10_ dopamine (DA) neurons, the latter of which project to regions like the amygdala, nucleus accumbens and prefrontal cortex to regulate executive decision-making based on cues associated with anticipated reward.

Food consumption is controlled and regulated *via* two circuits: the homeostatic and the hedonic energy balance circuitries. It is well known that the hypothalamus functions in both the modulation of energy homeostasis and feeding regulation. Four hypothalamic regions that contribute to this modulation are: the ventromedial nucleus (VMN), the arcuate nucleus (ARC), the LH, and the PVN. It has been shown that bilateral lesion of the VMN produced severe obesity – indicating that the VMN suppresses appetite and food intake; whereas large lesions of the LH led to death by inanition/starvation (20) – suggesting that the LH promotes food consumption and appetite. Similarly, the ARC and PVN are also known to regulate feeding as global ablation of the ARC (21) and lesions caudal to the PVN (22) resulted in morbid obesity and overeating. Within these regions, hormones like insulin and leptin released from the pancreas and adipose tissues, respectively, exert anorexigenic effects. In contrast, ghrelin released from the gastric mucosa exerts orexigenic effects. For instance, leptin suppresses appetite *via* the excitatory effect on anorexigenic POMC neurons in the ARC (23–25) that depolarizes these neurons and increases their firing rate through activation of transient receptor potential cation 5 (TRPC5) channels. On the other hand, leptin also exerts inhibitory effects on orexigenic neuropeptide Y (NPY)/agouti-related peptide (AgRP) neurons in the ARC (26, 27). Similarly, insulin also inhibits and hyperpolarizes NPY/AgRP neurons in the ARC, and it can inhibit SF-1 neurons in the VMN as well *via* the activation of ATP-gated potassium (K_ATP_) channels (28–30). Insulin can regulate POMC neuronal plasticity to control energy balance. For instance, the excitatory effect of insulin depolarizes and stimulates POMC neurons *via* the activation of TRPC5 channels (29, 31); whereas, its inhibitory effect results in hyperpolarization and inhibition of POMC neurons (24, 32–34). Interestingly, it is now known that the balance between excitatory and inhibitory of insulin-induced responses in POMC neurons is dependent on the levels of protein tyrosine phosphatase 1B (PTP1B) and T-cell protein tyrosine phosphatase (TCPTP) within these cells (34). In contrast to insulin and leptin, ghrelin promotes appetite *via* the excitatory effects on orexigenic NPY/AgRP neurons by depolarizing and increasing the firing rate while also inhibiting and suppressing firing of POMC neurons (35, 36). Satiety and hunger are two physiological responses that can directly reduce food consumption and promote feeding, respectively. If the homeostatic feeding regulates energy intake and expenditure based on survival and physiological needs, the hedonic feeding behavior modulates energy balance pertaining to reward-based or pleasure-driven food intake. The hedonic energy balance circuitry involves the mesolimbic dopamine (A_10_) neurons in its regulation of palatable food. These A_10_ dopamine neurons emanate from the ventral tegmental area (VTA) and project to several regions such as the nucleus accumbens (NAc), hippocampus, prefrontal cortex (PFC), and amygdala (37–39). Evidence accumulated over the past 20 years indicates that other neuropeptides like PACAP can regulate food intake in both homeostatic and hedonic circuitries. Indeed, the VMN PACAP neurons are ideally suited to do just that (**Figure 1**). In this review, we will focus on the effect of PACAP on the homeostatic and hedonic aspects of feeding. In this context, we aim to describe the *in vivo* and *in vitro* effects of both exogenous and endogenous PACAP in regulating energy intake and energy expenditure.

## The Homeostatic Energy Balance Circuitry Within the Hypothalamus

Several seminal lesioning studies conducted in rats and mice during the 50s and 60s denoted the importance of the hypothalamus in feeding regulation (20, 21). This provided a catalyst for future experiments leading to a better understanding of the hypothalamus as the fundamental nexus in controlling the whole-body energy balance within the homeostatic circuitry. The cross-communicating systems are conveyed by various hypothalamic nuclei and their associated neuronal populations, including those in the VMN, ARC, LH, the dorsomedial nucleus (DMN), and the PVN. These hypothalamic nuclei produce both anorexigenic and orexigenic signals to reduce and promote energy intake, respectively.

As mentioned above, the VMN has long been considered a central satiety center. SF-1 is a transcription factor encoded by the NR5A1 gene, and the SF-1 expressing neurons play crucial roles in the control of energy balance demonstrated by the studies of conditional knock-out mice (40–42). SF-1 can directly impact the VMN development such that the deletion of SF-1 or leptin receptor in SF-1 neurons leads to obesity in mice (42–44). In addition, the brain-derived neurotrophic factor (BDNF) as well as its neurotrophic receptor kinase 2 (NTRK2) receptor are also strongly expressed in the VMN, and the deletion of BDNF and its receptor leads to hyperphagia and obesity in humans and mice (45, 46). In contrast, the administration of BDNF either by central venous or peripheral venous results in body weight loss and food reduction through melanocortin 4 receptors (MC4R) signaling (47).

Excitatory input from VMN SF-1 neurons activates the anorexigenic POMC neurons, which leads to a reduction in energy intake and an increase in energy expenditure (48–50). POMC neurons in the ARC make up the melanocortin system of the hypothalamus. Alpha-melanocyte-stimulating hormone (α-MSH) consists of 13-amino acid residues and is derived from posttranslational modification of the POMC precursor peptide along with other bioactive peptides like the endogenous opioid β-endorphin (51–53). The ARC also houses another population of neurons that co-express orexigenic neuropeptide Y(NPY) and agouti-related peptide (AgRP) (54). POMC/CART and NPY/AgRP cells together with downstream target neurons expressing the MC4R (or MC3R) constitute the melanocortin system (55, 56). Studies have shown that α-MSH plays a critical role in the central nervous system (CNS) in suppressing food intake (55, 57) such that the MC4R knock-out mice exhibit obesity, hyperphagia, hyperleptinemia, and hyperglycemia (58). Similar results were observed with AgRP, a high-affinity competitive antagonist of MC4R, which directly inhibits POMC neurons and ultimately impedes the anorexigenic signaling of α-MSH to promote feeding and decrease energy expenditure (55, 56).

Previous studies have reported that μ-opioid agonists, inhibit POMC cells by inducing outward current and hyperpolarization (59–62) and promote appetite in rodents (63, 64) by physiologically antagonizing the effects of α-MSH on downstream melanocortin receptors (65). In contrast, β-endorphin knock-out male, but not wild-type male mice exhibit hyperphagia, hyperleptinemia, hyperinsulinemia, and greater adiposity (66). Additionally, the anorexigenic neuropeptide CART is co-expressed in an appreciable number of POMC neurons (67).

Of the orexigenic neuropeptides in the ARC, both NPY and AgRP can directly modulate energy balance. NPY/AgRP neurons inhibit POMC neurons *via* synaptic release of the inhibitory amino acid neurotransmitter gamma-aminobutyric acid (GABA) from these cells (68). NPY acts at five different receptors known as Y1, Y2, Y4, Y5, and y6; however, concrete evidence suggests that postsynaptic Y1 and Y5 receptors mediate the effects of NPY under *ad libitum*-fed conditions (69, 70). For instance, direct administration of NPY into specific hypothalamic sites like the VMN, PVN and LH (71–73), as well as injection into the lateral ventricle (72, 74–76) swiftly induces feeding responses in rats. Similarly, chronic central administration of AgRP reduces energy expenditure, induces food intake, and causes obesity (77–79). In congruence with these studies, Krashes and colleagues employed designer receptors exclusively activated by designer drugs (DREADDs) and chemogenetic approaches to acutely stimulate AgRP neurons through action on MC4R to induce feeding over a prolonged period as compared to the rapid stimulation of feeding by GABA and NPY – indicating that AgRP plays a role in a different phase of appetite regulation (80, 81). Despite these reports demonstrating the effects of NPY and AgRP as positive modulators of energy balance, some genetic studies have shown contradicting results as NPY- and AgRP- knock-out mice failed to show changes in feeding behavior and body weight (82–84). In contrast, several papers have indicated that AgRP ablation in adult mice eventually leads to death due to starvation but completely ineffective in neonatal mice (85). There is also a population of nociceptin/orphanin FQ (N/OFQ)-expressing neurons in the ARC. These cells are glucose- and leptin-sensitive, and their excitability is enhanced by acute exposure to a high-fat diet (HFD) (86). ARC N/OFQ neurons co-release GABA, and optogenetic stimulation of these cells inhibits neighboring POMC neurons *via* a combination of fast GABA_A_ receptor/mediated neurotransmission and activation G protein-gated, inwardly rectifying K^+^ (GIRK) channels (86, 87).

Besides the VMN and ARC, the LH also plays a crucial role in the regulation of energy balance (88). Two neurons that are abundantly expressed in the LH are melanin-concentrating hormone (MCH) and orexin. MCH has been recognized as a feeding stimulator in mammalian brain because MCH mRNA levels are not only upregulated by threefold in *ob/ob* (i.e., leptin deficient) mice under *ad libitum* fed, but MCH mRNA expression also increase under fasting condition in both *ob/ob* and normal mice (89). Several studies have demonstrated that intracerebroventricular (ICV) injections of MCH produce an increase in food intake in both rats (90, 91) and mice (92); whereas, mice with reduced MCH mRNA levels or disruption of the MCH1 receptor remain lean and hypophagic (93, 94). Orexin neurons, on the other hand, are found to have increased in mRNA expression under fasting conditions (95) and stimulation of these neurons increases both food intake and energy expenditure (96). Central administration of orexin not only promotes food consumption (95, 97), but also increases behavioral responses to food reward (98). Taken together, it could be said that neurons found within the LH, such as those containing orexin or MCH, can relay orexigenic signals. For example, orexin enhances GABAergic and diminishes glutamatergic inputs onto POMC neurons, which electrically silences these cells (99). On the other hand, endocannabinoids elicit its orexigenic effect by retrogradely inhibit GABAergic input onto MCH neurons in a leptin-dependent manner, which may account in part for the hyperphagic effect of these cells (100).

The PVN and DMN are also very crucial in the control of energy balance and regulation of food intake (74, 101, 102) and also play important roles in physiological processes including thermoregulation, stress, and appetite (103, 104). The PVN expresses high levels of MC4R and MC3R, and it receives innervation not only from the POMC and AgRP neurons from the ARC, but also from the NTS (105, 106). Direct injection of NPY or anti-NPY γ lgG into the PVN causes stimulatory or inhibitory effects on food consumption, respectively (107, 108). On the other hand, the DMN receives projections from the ARC and sends projections to the PVN and LH. Several studies reported that NPY expression in the DMN is increased in rodents with obesity (103, 109), and the increased NPY levels play a significant role in the development of diet induced obesity (DIO) as well as the regulation of thermogenesis (104).

Besides playing a significant role in the regulation of energy balance, these neurons are susceptible to the influence of peripheral hormones like insulin, leptin, ghrelin, and sex hormones. Leptin was first discovered by Zhang and his colleagues in 1994 which opened up new avenues of research in the regulation of body weight homeostasis and obesity (110). Leptin is synthesized primarily in mature adipocytes within white adipose tissue and acts as an anorexigenic hormone after being released into the bloodstream (110, 111). Leptin acts not only as an appetite suppressor, but also plays a role in stimulating metabolism and reducing excessive stored energy (112). There are several isoforms of leptin receptor (LepR) that have been cloned like Ob-Ra, Ob-Rb, Ob-Rc (113–116), but the long form (LepRb) is the most crucial to bring out the effects of leptin and is abundantly expressed in the hypothalamus (113). For instance, activation of LepRb *via* the Janus kinase (JAK)/signal transducer and activator of transcription (JAK/STAT) and phosphatidylinositol-4,5-bisphosphate 3-kinase (PI3K) signaling pathways depolarizes both the ARC POMC and VMN SF-1 neurons leading to ions entering and increase in firing *via* the activation of TRPC5 channels (23–25, 44). By contrast, the inhibitory effect of leptin on NPY/AgRP neurons is mediated by the activation of K_ATP_ channel; leading to hyperpolarization and decrease in firing (26). Studies of NPY/AgRP and POMC neurons from *ob/ob* mice as well as DIO guinea pigs and NR5A1-cre mice reveal that they undergo extensive synaptic plasticity under conditions of obesity; with the former getting more excitatory glutamatergic inputs and fewer inhibitory GABAergic inputs, and the latter receiving large number of GABAergic inputs and less glutamatergic input (49) (117); however, fasting can diminish the strength of the excitatory inputs onto POMC neurons (48). Previous reports have shown that a deficiency in leptin (i.e., *ob*/*ob*) or LepRb (i.e., *db*/*db*) not only causes hyperphagia and reduces energy expenditure in both humans and mice (118–120), but also results in morbid obesity in mice (114, 115).

Like leptin, insulin is known as an anorectic hormone in the hypothalamus and is synthesized in pancreatic β-cells. Insulin receptor (IR) is highly expressed in the hypothalamic areas that play a role in the regulation of energy balance (121, 122) and also colocalizes with POMC and AgRP neurons (123, 124). Though the absence of IR in these POMC and AgRP neurons did not significantly alter energy homeostasis, deficiencies in the ability of insulin to decrease hepatic glucose production and suppress adipocyte lipolysis were observed (124–126) – indicating that insulin not only acts as an anorexigenic hormone in the CNS but also plays an important role in glucose metabolism. While LepRb activity is mediated *via* JAK/STAT and PI3K signaling as well as TRPC5 and K_ATP_ channels, the inhibitory effects of insulin are mediated *via* similar yet distinct pathways. For instance, insulin inhibits NPY/AgRP (29, 127) and VMN SF-1 (28) neurons *via* the activation of K_ATP_ channels. On the other hand, insulin can exert both excitatory (via activation of TRPC5 channels) and inhibitory effects (via PI3K) on POMC neurons (24, 29, 32, 33, 128). The directionality of these effects depends on the levels of PTP1B and TCPTP expressed by POMC and NPY/AgRP neurons, which fluctuate with changes in energy status (34, 129, 130). The phosphatases PTP1B and TCPTP are highly expressed in the ARC and can directly regulate leptin and insulin signaling in POMC neurons such that PTP1B attenuates leptin activity while TCPTP decreases insulin signaling *via* dephosphorylation of JAK2 tyrosine kinase and IR. However, in NPY/AgRP neurons, TCPTP decreases only insulin signaling (129–131). Furthermore, deletion of both phosphatases in POMC neurons, or of TCPTP in NPY/AgRP neurons, from obese mice prevents DIO in animals fed a high-fat diet, increases energy expenditure, and enhances leptin and insulin signaling to promote weight loss (129, 130).

By contrast, ghrelin, acts as an orexigenic peptide and its circulating levels increase in response to negative energy balance. Within the ARC, ghrelin, the orexigenic peptide hormone, plays a crucial role in regulating energy intake and energy expenditure. To promote food intake, ghrelin acts on its growth hormone secretagogue receptor (GHSR), which is highly expressed in the ARC and is located near the median eminence, a site known to allow the swift access of circulating ghrelin (132). A growing body of evidence shows that peripheral administration of ghrelin selectively increases c-Fos expression in the ARC, and ghrelin fails to stimulate food consumption in ARC-ablated rats (133–135). Ghrelin regulates food intake and energy balance by stimulating the NPY/AgRP neurons to elicit an inward current coupled with depolarization and an increase in firing rate, and mice lacking NPY- and AgRP-expression are insensitive to the exogenous administration of ghrelin (35, 136). Similarly, ghrelin also increases NPY and AgRP mRNA expression (137, 138). The ghrelin-induced depolarization of NPY/AgRP neurons can occur directly through activation of postsynaptic sulfonylurea receptor 1/transient receptor potential melastatin 4 receptors as well as indirectly given that tetrodotoxin did not completely block the depolarization and decrease in input resistance caused by the peptide (139). The ghrelin-induced activation of NPY neurons causes an increase in inhibitory postsynaptic currents in POMC neurons, and the GABA_A_ receptor-mediated inhibition of POMC neurons is secondary to the excitatory effect on NPY/AgRP neurons – indicating that ghrelin is most likely stimulating the release of GABA from NPY neurons to induce this hyperpolarization (140). Indeed, the inhibition of both NPY Y1 and GABA_A_ receptors reverses the hyperpolarization of POMC neurons – suggesting that ghrelin stimulating the release of both NPY and GABA from NPY/AgRP/GABA nerve terminals (140). This increase in the firing of NPY/AgRP neurons enhances GABAergic input onto POMC neurons such that deletion of the vesicular GABA transporter in AgRP-cre mice blunts the ghrelin’s hyperphagic effect (68). This is an indicative that presynaptic GABA release from NPY/AgRP neurons is an essential mediator of ghrelin’s effect on food intake. Moreover, ghrelin enhances NPY’s feeding stimulant action and NPY’s effects on respiratory quotient (141). The fasting-induced increase in circulating ghrelin concentrations (142) is consistent with the increase in spontaneous firing rate in NPY/AgRP neurons from food-deprived mice compared to those from *ad libitum*-fed animals (143). Thus, either peripherally originated ghrelin or ghrelin neurons in the periventricular hypothalamus or ARC that send synaptic inputs onto various ARC neurons can regulate the activity of these cells by controlling the release of neuropeptides and inhibitory amino acid neurotransmitter (140, 144). Besides the ARC, the orexigenic effect of ghrelin may also be attributed to its actions in the PVN. Intranuclear administration of ghrelin into the PVN, where ghrelin GHSR-expressing cells are found (145), promotes appetite (146). Interestingly, it has been reported that blockade of cannabinoid CB1 receptors in the PVN not only stimulates fasting-induced hyperphagia but also increases ghrelin’s effect in *ad libitum*-fed rats (147). On the other hand, the orexigenic effects of both cannabinoids and ghrelin appear to involve the activation of AMP-activated protein kinase (AMPK) (148, 149). Lastly, it has been recently discovered that liver-expressed antimicrobial peptide-2 functions as an endogenous antagonist of the GHSR (150).

AMPK is known as a central regulator of lipid metabolism and of energy homeostasis. AMPK is a serine/threonine kinase comprising of two alternative α catalytic subunits (α1 and α2) as well as two regulatory β and γ subunits (151). Activation of AMPK is dependent on the cellular AMP : ATP ratio such that increasing in AMP level while decreasing in ATP level stimulates its activity (151, 152). According to Yan and colleagues, AMPK activity is regulated by direct allosteric activation and by activation loop phosphorylation, in which, ATP inhibits, and AMP triggers, AMPK in these mechanisms (152). As indicated, the orexigenic effects of ghrelin and cannabinoids are mediated by the activation of AMPK. There exists a ghrelin-cannabinoid interaction responsible for this, such that ghrelin requires an intact endocannabinoid system to promote food intake and fails to induce the same effect in CB1 knock-out mice (153). Furthermore, the AMPK-stimulating effect of cannabinoids was shown to be CB-1 dependent, which was blocked by rimonabant but had no effect in CB-1 knock-out animals (153). Taken together, the GHSR-mediated decrease in excitatory input onto parvocellular neurons in the PVN is mediated by the activation of AMPK as well as the increased production of endocannabinoids *via* subsequent activation of CB1 receptor (153, 154). Leptin and insulin also modulate the activity of energy-sensing AMPK, such that they inhibit both AMPK and its downstream targets in the hypothalamus (155, 156). Leptin does this by phosphorylation on serine491 of the α2AMPK subunit, which was found to be crucial for leptin’s effects on food intake and body weight (157). On the other hand, insulin does this by inducing direct phosphorylation *via* Akt (protein kinase B) on serine485 of the α1AMPK subunit, which was showed to inhibit threonine172 phosphorylation and thus promote cell survival and proliferation (158).

As mentioned above, K_ATP_ channels play a crucial role in the regulation of energy and glucose homeostasis (159, 160). They are present in most cell types, and are critical in regulating the membrane potential of neurons (161). The electrochemical gradient for K+ is such that its equilibrium potential is considerably more negative than the resting membrane potential of most neurons; therefore, the opening of K+ channels induces outward currents that result in K+ efflux and the hyperpolarization of the cell (160). Because K+ channels have a major role in setting the resting membrane potential, reducing action potential duration and decreasing firing rate, this indicates that these channels play a profound role in suppressing cell excitability (161). K_ATP_ (aka. Kir6) channels belong to a subfamily of the weak inward rectifier K+ channels that are classified into Kir6.1 and Kir6.2 subtypes (162). While Kir6.1 genes are mainly expressed in the mitochondria (163, 164), Kir6.2 subunits are involved in most functional K_ATP_ channels that are present in pancreatic β cells, cardiac muscle cells, smooth muscle cells, and all brain regions (162, 165, 166). These channels are negatively gated by ATP. For instance, a decrease in the ratio of ATP/ADP opens cardiac K_ATP_ channels and results in hyperpolarization, which stabilizes cardiac myocytes (162, 165). In contrast, an increase in the ratio of ATP/ADP in hyperglycemic conditions closes K_ATP_ channels of pancreatic β cells and leads to depolarization and insulin secretion (162). The antagonism of K_ATP_ channels can also be induced by tolbutamide, a K_ATP_ channel blocker that binds preferentially to the regulatory sulfonylurea receptor subunits SUR1 (165).

## The Hedonic Energy Balance Circuitry and the Mesolimbic DA Pathway

While the homeostatic-hypothalamic circuitry regulates energy balance based on physiological needs, the hedonic circuit modulates energy intake and expenditure based on food reward. A growing body of literature suggests that both the consumption of palatable foods and substance abuse converge on a shared pathway within the limbic system in humans and rodents alike to mediate motivated behaviors (167–169). This indicates that the consumption of palatable food involves the A_10_ neurons that originate from the VTA and project to the NAc, hippocampus, prefrontal cortex, and amygdala (37–39). The dopamine (DA) neurons were first discovered ~50 years ago and were classified anatomically based on the clustering of somata into three areas: the retrorubral area (A_8_), the substantia nigra pars compacta (SNc, A_9_), and the ventral tegmental area (VTA, A_10_) (170–172). These neurons share the rate limiting enzyme tyrosine hydroxylase and dopa decarboxylase, which catalyzes an irreversible decarboxylation reaction to produce dopamine (173). The VTA is made up predominantly of three types of neurons including GABAergic, glutamatergic and A_10_ DA cells. Beier and colleagues employed viral trans-synaptic tracing to demonstrate that the GABAergic and A_10_ DA neurons receive direct inputs from similar brain regions; the VTA GABAergic neurons, however, receive inputs mostly from the central amygdala (a region regulates emotions such as fear and aggression) and the anterior cortex (174). Consistent with this finding, stimulation of VTA GABAergic neurons induces place aversion and disruption in food consumption *via* the inhibition of DA activity; whereas, their inactivation promotes reward by enhancing the A_10_ DA signaling (175–177). In contrast, activation of DA neurons within the NAc promotes reward seeking behavior (178–181). In addition, A_10_ DA neurons have been shown to express vesicular glutamate transporters 2 (VGlut2), which reportedly enhances DA storage, neuronal growth and survival, the density of DA innervation of the NAc, as well as promotes the corelease of DA and glutamate, the latter of which induces a fast excitatory postsynaptic current (182–185). The knockout of VGlut2 gene, in contrast, reverses all the effects by abolishing glutamate release from A_10_ DA neurons and reducing the excitatory signal to the NAc (184, 185). In addition, local superfusion of glutamate at the VTA not only selectively activates A_10_ DA neurons but also enhances the excitatory synaptic signaling, whereas the superfusion of quinpirole, a D2 DA receptor agonist, increases the activation threshold of DA neurons and inhibits the excitatory synaptic event (182). This may be indicative that glutamatergic co-transmission plays a crucial role in conveying signals related to incentive salience. It is indeed the case as the activation of glutamatergic fibers in the anterior NAc shell causes a temporary cessation of feeding and promotes reward-seeking behavior (186).

As stated above, the A_10_ DA neurons receives direct input from other hypothalamic areas. For instance, it has been shown that a subpopulation of ARC POMC neurons project to the VTA to inhibit A_10_ DA neurons and induce hypophagia as a result (187). The local stimulation of VTA GABAergic neurons disrupts food intake and induces place aversion (175, 176), whereas photoactivation of LH GABAergic neurons projecting to the VTA instigates the opposite effect. This is because photostimulation of LH GABAergic neurons-VTA projection inhibits local VTA GABAergic neurons, increases DA release, and promotes social interaction and approaching, whereas photoactivation of VTA-projecting LH glutamatergic neurons reduces DA release and promotes avoidance (188). In addition, they noticed that the inhibition of LH GABAergic-VTA projection in food-restricted suppresses behavioral responses illustrated by significantly decreasing the time spent feeding and investigating the objects (188); however, the activation of LH GABAergic-VTA projection promotes feeding in sated mice (189). Within the LH, the two neuropeptides, orexin and MCH, have also been studied extensively to better understand how they modulate feeding and reward seeking behavior in the VTA. While both peptides project widely throughout the brain, orexin-expressing LH neurons also project to the A_10_ DA neurons to promote intake, but MCH-expressing LH neurons do not. For instance, orexin signals emanating from the LH to the VTA to promote neuronal activity by enhancing excitation *via* orexin receptors in the VTA and triggering DA neurons and thus promoting food intake (190, 191). On the other hand, MCH-expressing LH neurons project to the NAc to elevate food intake and reward reinforcement (168, 192, 193). Since neuropeptides from the LH project to the VTA and NAc to regulate energy balance, it is indicative that there must be a neural circuit conveying information from the homeostatic to the hedonic energy balance circuitry.

Interestingly, leptin and its receptors have been shown to modulate these two energy balance circuitries. Activation of VTA LepRb neurons decreases motivation for food reward and food intake in food-restricted mice (194), whereas the activation of VTA-projecting LH LepRb neurons, which form inhibitory synapses with non-DA VTA neurons and promote motivation for food intake (195). In addition, optogenetic activation of LH LepRb neuron-VTA projections drives appetitive learning while not affecting consummatory behaviors *in vivo* (196). Taken together, the neuropeptides and LepRb neurons in the LH can modulate energy balance within the hypothalamic-VTA-NAc circuits.

Hedonic eating is linked to the A_10_ system, which is known for its regulation of reward-related behaviors (197, 198) and its role in binge eating episodes (199, 200). Berridge and colleagues were one of the first teams to distinguish the role of A_10_ DA neurons in hedonic feeding by terming them as reward ‘liking’ and reward ‘wanting’ (201, 202). While ‘liking’ is associated with the hedonic pleasure triggered by a rewarding stimulus, such as highly palatable food, ‘wanting’ is related to incentive salience and is a mesolimbic-induced event that enhances reward-seeking (201, 202). In addition, ‘liking’ has been measured by observing changes in facial expressions provoked by taste stimulus in which sweet tastes are associated with lip smacking while bitter tastes showed a combination of face expressions like moving the brows, nose and face (203). In contrast, ‘wanting’ is established *via* neural interactions between the NAc, VTA, and amygdala, which is driven by both physiological state and a reward cue (201, 202). Though ‘wanting’ and ‘liking’ occur simultaneously, they can be psychologically and neurally distinguishable from one another and both are needed to achieve a full reward (202).

Pharmacological and genetic studies demonstrate that peripheral hormones such as leptin, insulin and ghrelin can act on the A_10_ DA systems to control food intake. For instance, leptin is involved in negative affective encoding within the DA reward system to regulate hedonic feeding behavior and energy balance. Several reports indicate that administration of leptin into the VTA decreases feeding, while adeno-associated virus (AAV)-mediated LepRb knockdown in the VTA leads to an increase in both food intake and the sensitivity to palatable foods (204, 205). Leptin does this by reducing the firing of DA neurons through the activation of an intracellular JAK/STAT pathway (204, 205). Like leptin, acute microinjection of insulin in the VTA induces long-term depression to suppress excitatory synaptic transmission onto A_10_ DA neurons and reduces food consumption (206–208). Insulin does this by suppressing alpha-amino-3-hydroxy-5-methyl-4-isoxazole (AMPA)-mediated excitatory post synaptic currents onto A_10_ DA neurons as well as inducing the activation of cannabinoid CB1 receptors, that in turn, inhibiting glutamate release (207). On the other hand, ghrelin binds to its GHSR to modulate DA release in the NAc and regulate hedonic feeding in a dose-dependent manner (209, 210). It has been reported that direct administration of ghrelin into the VTA or NAc triggers hedonic feeding. Ghrelin does this by triggering the cannabinoid CB1 signaling in VTA neurons to regulate release of GABA and glutamate, as well as increasing DA neuronal activity and turnover in a GHSR-dependent manner *via* the activation of Gq/phospholipase C (PLC)/diacylglycerol (DAG) protein signaling pathway and Gs/cyclic adenosine monophosphate (cAMP)/cAMP-response element binding protein (CREB) pathway (149, 209–211). Moreover, while the neuropeptide nociceptin/orphanin FQ (N/OFQ) is largely considered orexigenic through its actions within the homeostatic energy balance circuitry (212), it inhibits A_10_ DA neurons and attenuates binge feeding behavior when administered into the VTA (87). These findings reinforce the notion that A_10_ DA neurons are an important neural substrate in the regulation of incentive salience, motivated behaviors and hedonic feeding.

## Sex Differences in, and Activational Effects of Gonadal Hormones on, the Regulation of Energy Balance

Sex differences in the context of energy homeostasis have been studied extensively. Some reports have shown that men have a higher prevalence of obesity in early adulthood than their age-matched female counterparts, but this gender difference tapers off as reproductive senescence is approached (213, 214). Other studies have reported that women exhibit a higher chance of developing eating disorders and extreme obesity (215, 216). Sex differences exist in the regulation of energy balance as men and women not only crave different kinds of food but also show diverse changes under the condition of negative energy balance. For instance, previous studies reported that women experience more frequent episodes of state craving (217–219) while men report 15.6% fewer cravings for sugar-sweetened drinks (219, 220). Compared to men, women were reported to exhibit greater neural reactivity in the orbitofrontal cortex and insula in response to highly palatable food under food deprivation (221). This is congruent with other lines of evidence depicting that women have a reduced ability to control food desire, higher cortical and limbic activation when presented with visual, olfactory, gustatory cues, and increased susceptibility to episodes of food-craving compared to men (222–225). Sex is also thought to be one of the main determinants for susceptibility to food-related disorders, including binge eating disorder (226, 227). Indeed, binge-prone female rats endured high levels of foot shock for access to Oreo cookies in comparison to males, confirming the ‘continued use despite negative consequences’ criteria for substance dependence (228). Similarly, the N/OFQ-induced suppression of binge feeding is greater in females than in males, and more robust in obese subjects than in lean ones (87). Consistent with these findings, Yu and co-workers assessed sex differences using self-reported of the Yale Food Addiction Scale and Eating Attitude Test, and they reported that female college students were 3 times more likely to exhibit disordered eating behaviors that was positively associated with food addiction regardless of weight status (229).

The role of sex hormones, especially the circulating estrogen levels, play in an important role in feeding regulation as feeding decreases during the estrous stage when serum estrogen levels are at the highest (230). Consistent with this result, the administration of estrogen, either centrally or peripherally, greatly reduces food intake and decreases body weight (230–235). 17β-estradiol (E_2_) is the predominant form of estrogen in the non-pregnant state. E_2_ diffuses through biological membranes and interact with two well-known estrogen receptors (ER): ERα and ERβ. ERα is highly expressed in the ARC and the VMN, whereas ERβ is abundantly expressed in other regions of the brain but scarcely expressed in the ARC (236). It has been shown that ERα is involved in E_2_-mediated effects on body weight and food intake, as mice with ERα deficiency have increased body weight and heightened food consumption in both male and female mice (237). Estrogenic actions are mediated by two different cellular responses; one is characterized by changes in gene transcription when estrogen binds to nuclear ERs (aka. ERα and ERβ), and the other involves rapid signaling initiated by E_2_ binding to membrane-bound ERα, ERβ or the Gq-coupled membrane ER (Gq-mER), which activates second messengers signaling molecules such as PLC and protein kinase A (PKA) (238–240). Gq-mER has been shown to induce opposite effects on POMC and NPY/AgRP neurons. For instance, the activation of Gq-mER by E_2_ or the nonsteroidal compound STX uncouples μ-opioid, CB1, nociceptin opioid and GABA_B_ receptors from GIRK channels in POMC neurons; resulting in an increase in membrane excitability illustrated by diminished outward currents and hyperpolarizations in these cells (240–243). In contrast, the activation of Gq-mER by E_2_ or STX enhances the ability of GABA_B_ receptors to activate GIRK channels in NPY/AgRP neurons thus eliciting robust but reversible inhibitory outward currents (244). Interestingly, there is evidence to suggest that kappa opioids are involved in the weight gain associated with the hypoestrogenic state (245). Together, these findings confirm that the estrogenic actions mediated *via* ERs and Gq-mER play a crucial role in regulating energy homeostasis

As mentioned, ERα is abundantly expressed in the ARC, which houses both POMC and NPY/AgRP neurons. A growing body of evidence has reported that the effects of E_2_ on feeding largely occur through multifaceted actions on synapses involving these cells (238, 244, 246, 247), while E_2_ in the VMN regulates brown adipose tissue (BAT) thermogenesis (246, 248, 249). In addition, central administration of E_2_ reduces food intake in controls but not in AgRP knock-out mice (250). Furthermore, E_2_ dampens the refeeding response and suppresses c-Fos activation in NPY/AgRP neurons of fasted female mice (250) – suggesting that estrogen may regulate NPY/AgRP neurons indirectly *via* presynaptic neurons that express ERα. E_2_ might also do this by influencing other peripheral hormones, as E_2_ was reported to inhibit ghrelin’s orexigenic effect (251); ghrelin, in turn, regulates food intake and energy balance in large part through its effects on NPY/AgRP neurons. E_2_ has been shown to affect the M-current flowing through KCNQ channels, which is a voltage- and time-dependent, non-inactivating outward K^+^ current that is often targeted by G-protein-coupled receptors (252). The KCNQ 2, 3 and 5 subunits are abundantly expressed in the NPY/AgRP and POMC neurons. Fasting attenuates M-current and increases the excitability in NPY neurons by reducing the mRNA expression of KCNQ 2 and 3 subunits, whereas E_2_ escalates M-current and reduces the excitability of NPY neurons to decrease food intake by increasing the expression of KCNQ5 while not affecting KCNQ2 and KCNQ3 subunits (252). In addition, targeted knockout of KCNQ3 has been shown to reduce M-current in NPY neurons which correlated to an increase in body weight of high-fat diet (HFD)-fed mice but did not affect food consumption (253). In contrast, the inhibition of M-current has been shown to enhance the activity of POMC neurons in *ad libitum* chow-fed mice demonstrated by the reduction of food intake and the control of glucose homeostasis (254, 255). The Kisspeptin/Neurokinin B/Dynorphin (KNDy) neurons in the ARC also plays an important role in controlling of energy balance as optogenetic stimulation of the kisspeptin neurons directly excites POMC neurons and induces a depolarization (256, 257). In congruence with these findings, E_2_ has been shown to increase the ARC KNDy neuronal activity *via* ghrelin-induced inhibition of the M-current in female mice (258), and also enhances glutamate release from KNDy neurons that excites POMC neurons and inhibits NPY/AgRP neurons *via* subsequent activation of metabotropic group I and group II/III receptors, respectively (259). This, in turn, likely contributes to the anorexigenic actions of the steroid. Finally, ERs are also found within the VTA, as intra-VTA injection of E_2_ significantly reduced sucrose-seeking behaviors within an hour after injection (260).

In contrast to estrogen, testosterone in males rapidly increases food intake caused by the activation of cannabinoid CB1 receptors that leads to the decrease in glutamatergic input onto POMC neurons (261, 262). Testosterone induces the increase in endocannabinoid tone *via* the activation of AMPK, which leads to suppression of glutamate release at VMN SF-1/ARC POMC synapses *via* the upregulation of DAG lipase-α (261, 262). Interestingly, these effects are intensified in obese males due to reduced PI3K signaling in the ARC (49, 128). In addition, the appetite-stimulating effect of N/OFQ, as well as its ability to inhibit POMC neurons *via* activation of GIRK channels and presynaptically inhibit glutamatergic input onto these cells, is greater in males than their female counterparts (263).

## Dynamic and Pleiotropic Involvement of PACAP in the Regulation of Homeostatic and Hedonic Energy Balance Circuits

Pituitary adenylate cyclase-activating polypeptide (PACAP) was first isolated in 1989 from ovine hypothalamus (264). It has since been revealed that PACAP is highly conserved among vertebrates in terms of its amino acid sequence, which remained almost unchanged during an evolutionary period of ~700 million years (265–267); indicating its critical role in a number of different physiological responses (266, 268, 269). PACAP presents in two isoforms: a neuropeptide with 38 amino acid residues named PACAP_1-38_ or a C-terminally truncated version with 27 residues called PACAP_1-27_ as well as PACAP-related peptides (266, 267). PACAP exhibits high homology to vasoactive intestinal peptide (VIP), and is considered to belong to the VIP/secretin/growth hormone-releasing hormone/glucagon superfamily (268). PACAP is localized throughout the central and peripheral nervous system (CNS - PNS); thus, PACAP is equipped to act as a neurotransmitter, hormone, as well as a trophic factor in various cell types (268). In the rat brain, the highest concentration of PACAP can be found in the hypothalamus (270), which helps regulate many metabolic processes and activities of the autonomic nervous system. PACAP exerts a wide range of biological effects such as the regulation of hormone secretion, the control of neurotransmitter release, as well as neurotrophic and neuroendocrine actions (266, 269). There are two classes of PACAP receptors. The type I receptor is termed the PACAP-specific receptor (PAC1R), which has high binding affinity and selectivity for PACAP. On the other hand, type II receptors were originally called VIP-PACAP 1 and 2 receptors but were later reclassified as VPAC1R and VPAC2R, and they exhibit high binding affinity for both PACAP and VIP (267). The PACAP/PAC1 receptor system is distributed throughout the central nervous system, and is highly expressed in hypothalamic nuclei including the ARC, DMN, VMN, and PVN (266, 268, 271, 272).

**Figure 2:**
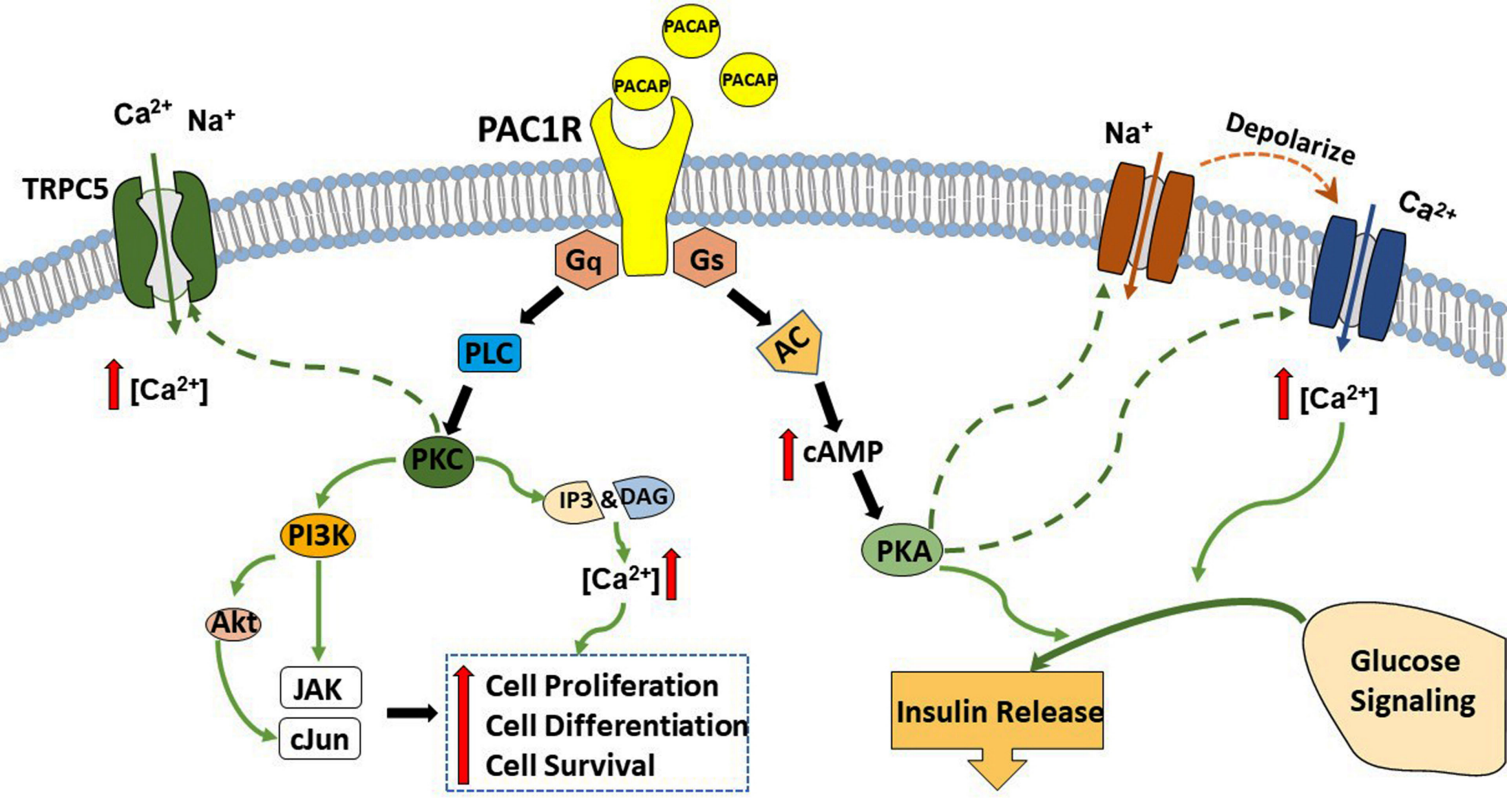
A schematic summary of PACAP signaling cascades. PACAP exerts its effects *via* interaction and activation of the PAC1 receptor. This receptor couples to both G_s_ and G_q_. Upon PAC1 receptor stimulation activation of G_s_ and subsequently adenylyl cyclase (AC) triggers the production of cyclic adenosine monophosphate (cAMP), which augments protein kinase A (PKA) phosphorylation and feeds into glucose signaling and insulin release. In contrast, the coupling with G_q_ activates phospholipase C (PLC) and boosts the production of inositol triphosphate (IP)_3_ and diacylglycerol (DAG), which leads to the activation of protein kinase C (PKC) and increases intracellular Ca^2+^ to promote cell survival, cell proliferation, and cell differentiation.

There are two separate pathways that can be stimulated upon PAC1 receptor activation; the G protein coupled receptors Gs and Gq pathways. The insulinotropic effect of PACAP is triggered *via* the Gs pathway. PACAP binds to PAC1 receptor and activates Gs, which turns on adenylate cyclase (AC), leads to the production of cAMP, and activates PKA. PKA, in turn, opens Ca^2+^ and Na^+^ channels and augments glucose-induced insulin secretion (269). In addition, activation of the Gs by PACAP also leads to increase in firing and depolarize the membrane potential of magnocellular neurons in rats brain slices (273–275). PACAP also has a neuroprotective effect which is accomplished through the Gq pathway. Briefly, PACAP binds to the PAC1 receptor and stimulates Gq, which activates PLC. PLC generates inositol triphosphate (IP3) and DAG, which boosts protein kinase C (PKC) activity to stimulate cell proliferation, cell survival, cell differentiation, as well as activate TRPC5 channels (50, 268). Known for its pleiotropic effects, PACAP can act differentially in multiple brain regions to stimulate signaling factors and modulate several ionic currents. The signaling pathways of PACAP will be summarized in the schematic shown in **Figure 2**.

PACAP and VIP are known to exert both neurotrophic and neuroprotective effects (268). These effects are stimulated through the activation of different signaling pathways. Several reports suggest that PACAP can restore not only dopaminergic transmission but also hippocampal cholinergic transmission (276–278). This is indicative that PACAP may be a promising therapeutic agent to fight against cognitive decline in Parkinson’s and Alzheimer’s disease. Parkinson’s disease is a neurodegenerative disorder that is second most common after Alzheimer’s disease, and a cure has yet to be found. Along these lines, PACAP exhibits promising neuroprotective properties as it has been shown to rescue DA neurons from neurodegeneration and improve locomotor function in rat parkinsonian models (278).

As stated, the hypothalamic areas such as the ARC, VMN and PVN play an important role in the regulation of energy expenditure, energy intake, and thermogenesis. Furthermore, these regions highly express PACAP and its PAC1 receptor, suggesting that PACAP must also play a role in these processes (268, 270, 279). Studies that employed laser microdissection revealed a number of VMN-enriched genes; including that encoding for PACAP, the expression of which is diminished in SF-1 knock-out mice (280). PACAP is also found to colocalize with SF-1 within the VMN (281). Administration of PACAP has been shown to notably diminish food consumption either by intraperitoneal (IP) injection (282, 283), ICV (271, 284, 285), intra-PVN (272), intra-VMN (272, 286, 287), intra-VTA (288), or intra-ARC (50, 212) under *ad libitum*-fed, HFD-fed, and fasting conditions. Several studies found that PACAP given ICV or intra-VMN in CD-1 mice and Sprague-Dawley rats increases locomotor activity, raises in O_2_ consumption and core body temperature (272, 281, 286) as well as elevates uncoupling protein 1 mRNA expression while reducing interscapular brown adipose tissue level (272). In contrast, intra-PVN administration of PACAP is without effect on locomotor activity and core body temperature (272).. Interestingly, ICV injection of PACAP at higher doses reduced locomotion in C57BL/6J mice (289) – suggesting the difference in doses of this peptide may affect the final results when used in different species. Similarly, intra-ARC administration of PACAP significantly increases O_2_ consumption and metabolic heat production while decreasing in respiratory exchange ratio (RER), and these effects are attenuated in DIO male mice (50). In ovariectomized (OVX) females, PACAP also significantly decreases cumulative energy intake, and increases O_2_ consumption, CO_2_ production, and metabolic heat production. The anorexigenic effect, but not the catabolic effects, of PACAP in OVX females are potentiated by E_2_ (50). Consistent with this finding, McMillan and colleagues reported that PACAP is likely involved in the melanocortin system regulation of sympathetic nerve activity that triggers thermogenesis. Although treatment with melanotan II, a melanocortin receptor agonist, did not affect body weight, white adipose tissue and lipid content of brown adipose tissue, melanotan II partially rescued the impaired thermogenic capacity of PACAP deficient mice under cold-acclimated condition compared to the controls (290). The appetite-suppressing and metabolic-enhancing effects of PACAP administered into the mediobasal hypothalamus are most likely due PAC1 receptor-mediated excitation of POMC neurons *via* activation of TRPC5 channels. This effect can be attributed to a G_q_-coupled signaling pathway involving PI3K, potentiated by E_2_ in females *via* activation of ERα and G_q_-mER, and diminished by DIO in males (50). Optogenetic stimulation of VMN PACAP neurons elicited the same excitatory effect in POMC neurons, which was again augmented by E_2_ in females (50). Similarly, IP injection of PACAP prior to the dark cycle decreases food intake in wildtype but not PAC1 receptor fl/fl mice and it does this in a dose-dependent manner (283). Furthermore, only the highest dose of PACAP [10 μM] consistently and significantly suppressed bout duration, bout frequency, meal size, time spent in feeding, and rate of consumption during the first 8-hour after injection compared to the vehicle, while the lowest dose [100 nM] only significantly attenuated rate of consumption (283) – illustrating that these effects were achieved *via* the coupling of PACAP/PAC1 receptor system.

**Figure 3:**
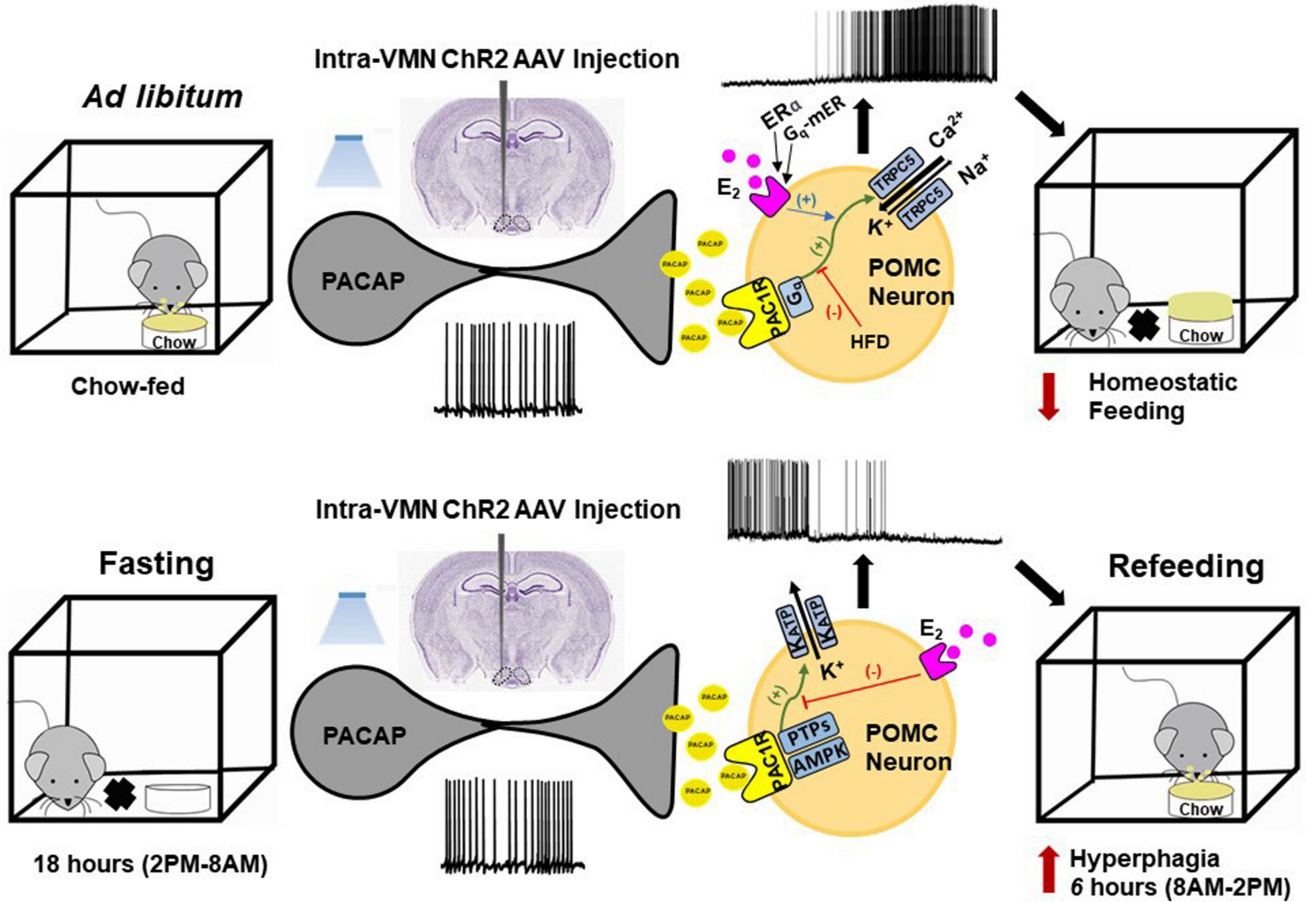
Schematic overview of the energy status-dependent plastic changes in PAC1 receptor-mediated signaling at VMN PACAP/ARC POMC synapses. *Top row*, Under *ad libitum*-fed conditions, optogenetic stimulation of VMN PACAP neurons and subsequent release of PACAP stimulates PAC1 receptors in ARC POMC neurons, which in turn activates TRPC5 channels *via* G_q_-mediated signaling. This depolarizes the POMC neurons and increases their firing, and ultimately decreases homeostatic feeding. These effects are potentiated by membrane-initiated estrogenic signaling *via* ERα and G_q_-mER in females, and diminished by DIO in males. *Bottom row*, Under fasting conditions, PAC1 receptor/effector coupling in POMC neurons switches from TRPC5 channels to K_ATP_ channels *via* upregulation of AMPK and protein tyrosine phosphatases (PTPs) like PTP1B and TCPTP. This hyperpolarizes the POMC neurons and suppresses their firing; ultimately enhancing the rebound hyperphagia that occurs during refeeding.

How PACAP exerts its anorexigenic or orexigenic effect to regulate the homeostatic energy balance depend not only on its site of injection but also on the anatomical location of particular PACAP neuronal populations as well as the ambient energy status, with *ad-libitum* (chow or HFD) feeding reflecting a more positive energy balance relative to fasting. Food-restriction (i.e., fasting) affects the levels of endogenous PACAP. Some have reported that PACAP expression is elevated in the hypothalamus of food-deprived rats as well as in chickens; suggesting that PACAP acts as a regulator of food intake (291). This was confirmed in mice in a sex differentiated manner such that hypothalamic PACAP levels increased to a greater extent in male than in female mice with food deprivation, while they decreased to a greater extent in female than in male mice under water deprivation (292). The result enhances the idea that male and female PACAP systems react differently under food deprivation, which may explain their differences in the choice of food and amount of food consumption in response to starvation (221). Still, others report that positive energy states upregulate PACAP levels in the VMN of mice (281), food restriction reduces the PACAP mRNA expression in mice in both the hypothalamus and more specifically the VMN (271, 281). In addition, food-restricted mice show low levels of POMC and PACAP mRNA expression coupled with high NPY mRNA expression, and ICV injection of PACAP decrease energy intake after 30-min of refeeding (271, 281). Interestingly, and yes paradoxically, we found that fasting completely reversed the anorexigenic effect of an intra-ARC injection of PACAP by significantly increasing the cumulative energy intake of wildtype mice, which is greatly reduced by E_2_ in OVX female mice (212). This reversal was due to a switch in the polarity of the response in POMC neurons from excitatory to inhibitory caused by a shift in the coupling of PAC1 receptors from TRPC5 channels to K_ATP_ channels brought on by upregulated AMPK as well as PTP1B and TCPTP in these cells (212). These fasting-induced changes in PAC1 receptor-mediated signaling in ARC POMC neurons is highlighted in **Figure 3**.

In other studies, researchers found that PACAP-deficient mice showed reduction in food intake, carbohydrate intake, and NPY mRNA expression (293), suggesting that endogenous PACAP affects carbohydrate consumption *via* the orexigenic effect of NPY/AgRP neurons as PACAP and its receptors including PAC1R and VPAC2R are highly expressed on NPY neurons (294). In congruence with these findings, PACAP neurons in the PVN provide excitatory input onto NPY/AgRP neurons that can drive homeostatic feeding (295). Additionally, PACAP knock-out mice show reduced nocturnal and daily food intake as compared to the controls, but diurnal intake was increased; indicating that food intake may be dependent on time of the day for these PACAP knock-out mice (296). In addition, PACAP knock-out mice exhibit increased POMC mRNA expression and decreased AgRP mRNA level (296), and AgRP levels were higher in the wildtype compared to PACAP knock-out mice. Furthermore, ICV administration of PACAP_6-38_ lead to a reduction in food intake, body weight, and AgRP expression in wildtype mice, while no changes were observed in POMC expression (296). This is indicative that PACAP can act as either an orexigenic or anorexigenic neuropeptide in the hypothalamus, and PACAP is capable of regulating the release of the peptides α-MSH and AgRP. As a neuropeptide that is intimately involved in energy homeostasis, PACAP may also be capable of regulating the release of other peptides like leptin, insulin, and ghrelin. This was indeed the case under the fasting state and postprandially where insulin and leptin expression levels were reduced in PAC1 knock-out mice, while ghrelin levels were greatly elevated by overnight fasting and postprandially in PAC1 knock-out mice compared to the wildtype (283). Additionally, evidence suggests that PACAP may mediate the anorexigenic effect of leptin (297). As with POMC neurons, leptin depolarizes VMN SF-1/PACAP neurons and increases their firing rate (44). Leptin injection into the VMN reduced food intake compared to saline-treated animals and the effect was completely blunted by PACAP_6-38_; however, administration of PACAP_6-38_ per se into the VMN showed no effect on either food consumption or body weight (297). This gives the indication that leptin may exert its effect *via* PAC1R activation in the VMN. Furthermore, they found not only that PACAP expression is colocalized with BDNF mRNA, but PAC1R expression was also co-expressed with BDNF and LepRb. With this information, they administered PACAP in the VMN and observed STAT3 phosphorylation as well as increased BDNF and suppressor of cytokine signaling 3 mRNA levels – just like with LepRb activation (297). Again, these effects of leptin and PACAP were blocked by PACAP_6-38_; suggesting that PACAP is downstream of leptin and mediating leptin’s effect on energy balance (296). Indeed, this may be how leptin activates the anorexigenic VMN PACAP/ARC POMC circuit under normophysiologic conditions (50, 212). Taken together, PACAP is capable of exerting dynamically pleiotropic actions within the homeostatic energy balance circuitry that include mediating the anorexigenic actions of leptin and regulating the activity of POMC and NPY/AgRP neurons as well as other peptide systems such as α-MSH, AgRP, and leptin.

The BNST plays a crucial role in regulating anxiety caused by long-term threats, and it is also important in mediating stress-induced anorexia and associated weight loss (298–300). The term “stress” refers to processes involving appraisal, perception, and response to stimuli or noxious events (301). Stress is manifested through two different pathways: the sympathetic adrenal medullary system, which releases catecholamines during periods of acute stress, and the hypothalamic-pituitary-adrenal (HPA) axis (302). The infusion of PACAP in the posterior BNST in both female and male Sprague-Dawley rats mimics weight loss induced by long-term stress exposure (300). Interestingly, the effect was not seen with PACAP administration ICV or in the anterior BNST – suggesting that PACAP exerts its effect only in the posterior BNST.

Given the role of PACAP in the posterior BNST, and the significance of the BNST in regulating food intake and the response to stress, this suggests that the PACAP/PAC1 receptor system may also be involved in stress-induced eating disorders. Prolonged stress increases glucocorticoid secretion, which can work synergistically with insulin to promote abdominal fat deposition, decrease HPA axis activity, and consequently affect energy homeostasis and eating behavior (303). It has been shown that chronically stressed rats tend to eat more under acute stress conditions and show a preference to consume palatable food (304, 305). Similarly, stress produces a greater intake of palatable food in overweight or obese individuals as compared to lean individuals (306, 307), and promotes food-seeking even in the absence of hunger and homeostatic need for calories (308). Stress-induced eating may contribute to the development of obesity (304), and may also adversely impact meal pattern and food preference (304). As mentioned above, stress increases the consumption of palatable food (304, 305), which might be an indicator of elevated DA release from A_10_ DA neurons. Indeed, DA release in the dorsal striatum is enhanced in obese, binge eating disorder (BED) participants as compared to non-BED obese participants during exposure to food cues (309, 310). The hallmark of BED is the excess consumption of food (311), which could result in an elevated body mass index (BMI). In addition, BED is significantly and strongly associated with severe obesity (BMI > 40) (312, 313). Taken together, stress increases vulnerability to binge-eating behavior and promotes irregular eating patterns, and these effects may be intensified in overweight and obese individuals.

**Figure 4:**
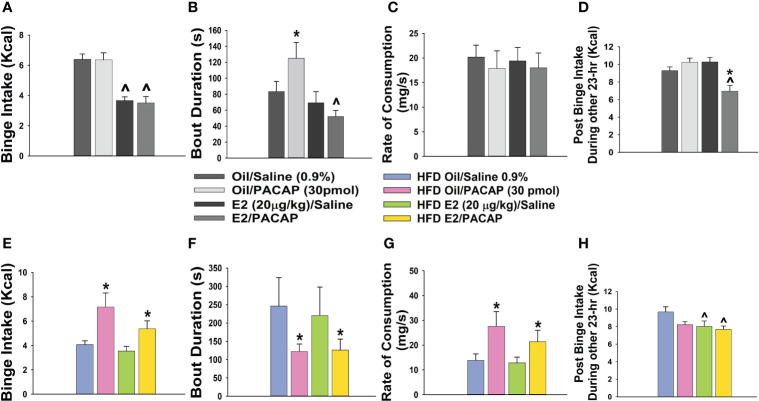
Dichotomous effects of intra-VTA PACAP on binge feeding in OVX, lean vs obese, sesame oil- vs EB-treated female mice. In lean chow-fed mice, EB (20 μg/kg; s.c.) but not PACAP (30 pmol) decreases binge intake **(A)**, and significantly reduces bout duration in PACAP-treated animals **(B)**. Neither E2 nor PACAP have any effect on rate of consumption **(C)**. EB and PACAP work synergistically to decrease chow intake during the remaining 23 hours **(D)**. In obese HFD-fed animals given a one-week respite prior to implementation of the binge paradigm, PACAP increases binge intake in both oil- and EB-treated females **(E)**, which is associated with decreased bout duration **(F)** and increased rate of consumption **(G)**. EB reduces homeostatic feeding during the remaining 23 hours **(H)**. Bars represent means and lines 1 SEM. ^, p < 0.05; with respect to sesame oil; *, p < 0.05; with respect to saline; repeated measures, multi-factorial ANOVA/LSD **(A–H)**; n = 6 for all treatment groups.

**Figure 5:**
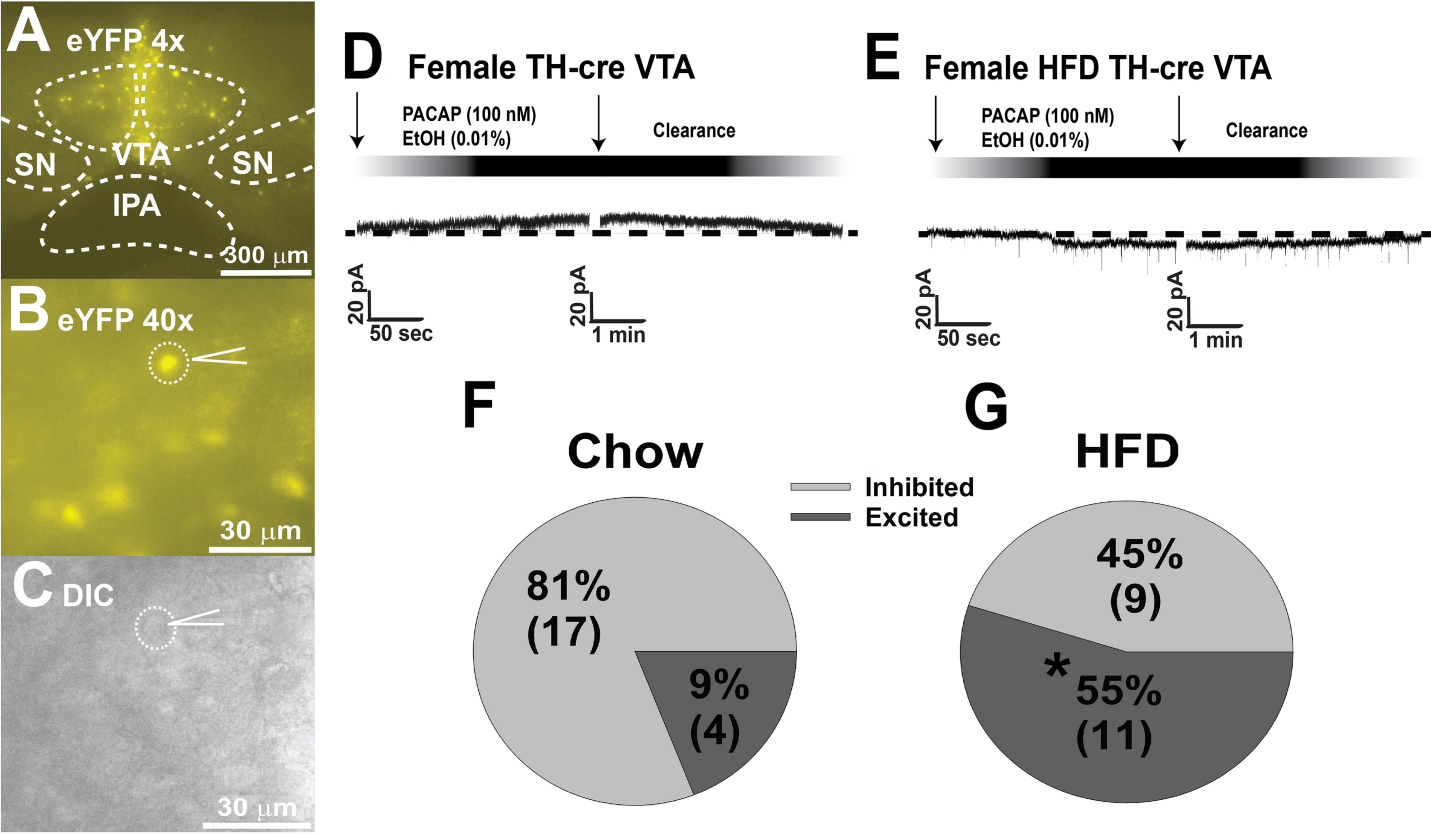
DIO-induced alterations in the response profile of A_10_ DA neurons from OVX female TH-cre mice to the postsynaptic effect of PACAP. **(A, B)** enhanced yellow fluorescent protein signal from A_10_ DA neurons in VTA slices captured at 4X and 40X. **(C)** differential interference contrast image of the recorded A_10_ DA soma seen in **(B)**. **(D, F)** PACAP (100 nM; n = 17) produces a reversible outward current in the considerable majority (17/21; 81% vs males): of A_10_ DA neurons from lean, chow-fed females, which is very similar to the percentage of PACAP-inhibited A10 DA neurons seen lean, chow-fed males (21/24; 87%). **(E, G)** Conversely, PACAP (n = 11) exerts a more heterogenous response in A_10_ DA neurons from obese, HFD-fed females, with the majority (11/20; 55%) of them being an excitatory inward current. Arrows indicate where I/Vs were conducted. *, p < 0.05; with respect to chow-fed controls, Chi-squared test.

**Figure 6:**
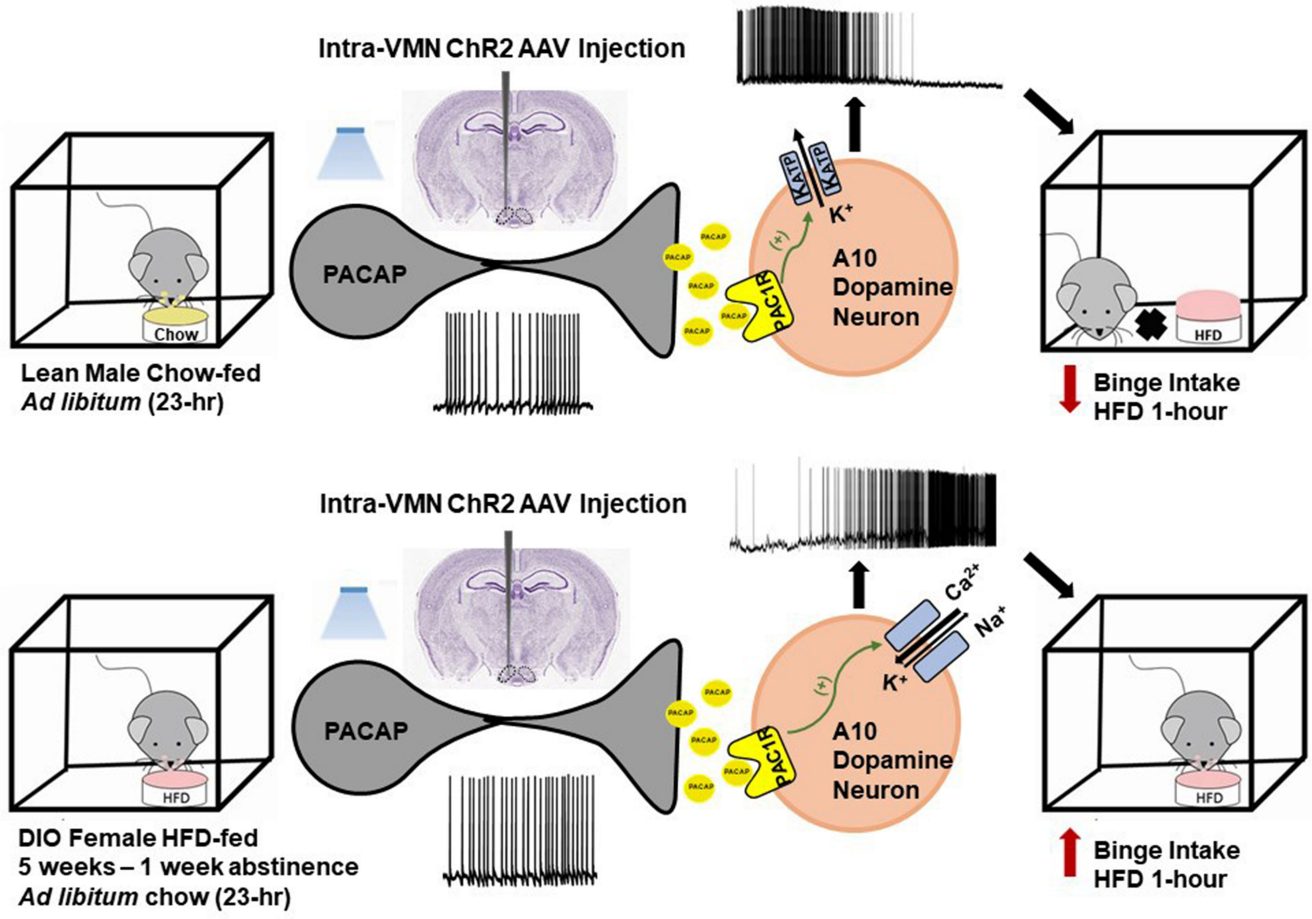
Schematic representation of the energy status-dependent changes in PAC1 receptor/effector coupling at VMN PACAP/VTA A_10_ DA synapses. *Top row*, In VMN PACAP neurons from lean chow-fed males, optogenetic stimulation leads to activation of PAC1 receptors that couple to K_ATP_ channels in A_10_ DA neurons; causing hyperpolarization cessation of firing. This in turn attenuates binge feeding brought on by brief intermittent exposure to HFD. *Bottom row*, In VMN PACAP neurons from DIO females, optogenetic stimulation leads to a far more heterogeneous response; with the majority of A_10_ DA neurons being excited *via* activation of mixed cation channels that ultimately potentiates binge feeding behavior.

In addition to its critical functions within the homeostatic energy balance circuitry, PACAP can also regulate the consumption of palatable food *via* the hedonic energy balance circuitry. PACAP effects within the hedonic circuit were demonstrated when Hurley and coworkers microinjected PACAP into the NAc, which then mimicked the actions of GABA agonists to reduce hedonic feeding as well as hedonic drive (287, 314). By contrast, while intra-VMN microinjection of PACAP replicates the AMPA-induced reduction in homeostatic feeding, it was without effect on hedonic feeding (287). Similarly, we have shown that PACAP administered into the VTA attenuates the binge-like consumption of palatable food (limited to one hour/day for five consecutive days) in lean male wildtype mice (288). This can be attributed to the PAC1 receptor-mediated activation of K_ATP_ channels in A_10_ DA neurons following bath application of PACAP, which results in membrane hyperpolarization and the cessation of firing. This inhibitory effect is mirrored by optogenetic stimulation of VTA-projecting VMN PACAP neurons (288). Surprisingly, intra-VTA PACAP is without effect on binge feeding behavior in either estradiol- or vehicle-treated OVX wildtype females (288) (**Figure 4A**). Estradiol did abrogate the PACAP-induced increase in bout duration (**Figure 4B**), and homeostatic feeding observed during the remaining 23 hours of the day was reduced in estradiol- and PACAP-treated OVX females (**Figure 4D**). However, neither E2 nor PACAP have any effect on rate of consumption (**Figure 4C**). Given the links between obesity and BED, we then investigated whether PACAP could suppress the rampant binge feeding observed in DIO OVX females (87). Unexpectedly, intra-VTA PACAP increased rather than decreased binge feeding in these animals (**Figure 4E**). This is associated with a decrease in bout duration (**Figure 4F**), and a sizable increase in the rate of consumption (**Figure 4G**). We followed this up with *in vitro* recordings in mesencephalic slices from lean, chow-fed and DIO, HFD-fed OVX TH-cre mice. We found that the PACAP-induced change in the excitability of A_10_ DA neurons switched from predominantly inhibitory in recordings from lean, chow-fed animals to excitatory seen in the majority of cells from DIO, HFD-fed animals (**Figure 5**), and this is illustrated schematically in **Figure 6**.

## Concluding Remarks

Extensive ongoing research for the past several decades has identified many of the major players comprising the circuits involved in homeostatic and hedonic energy balance regulation. We now understand that the components of the homeostatic energy balance circuits are deeply interconnected and ultimately converge at sites of integration within the PVN. We also know that peripheral hormones like leptin, insulin and ghrelin impart energy status-dependent signals to the neural substrates of these circuitries; changing their excitability in ways that profoundly impact appetitive behavior and satiety. More importantly, there is a growing appreciation for how connections between the hypothalamus and VTA as well as the NAc allow for cross-talk between the homeostatic and hedonic energy balance circuitries that coordinates nutrient- and palatability-based energy intake.

PACAP has emerged as another major player in the regulation of energy homeostasis. A considerable body of evidence accumulated over the past 30 years indicates that the PACAP/PAC1 receptor system exerts perplexingly pleiotropic actions in regulating both the homeostatic and hedonic energy balance circuitries. The anorexigenic and orexigenic effects of PACAP are dependent on site of injection, the anatomical location of the PACAP neuronal populations (i.e., VMN vs PVN), and strongly influenced by the ambient energy status not to mention the animal model and strain used in any given study. We have demonstrated that PACAP reduces homeostatic feeding and increases energy expenditure in lean, sated chow-fed animals, due largely to its excitatory actions on ARC POMC neurons. These appetite-suppressant and catabolic effects are diminished by DIO in males, whereas the anorexigenic (but not the metabolic) effect is potentiated by E_2_ in OVX females, and completely reversed under negative energy balance upon fasting. On the other hand, we have shown that PACAP dampens hedonic binge feeding through its inhibitory actions on A_10_ DA neurons in the VTA. This effect is sexually differentiated in that it occurs in lean, otherwise chow-fed males but not OVX females, and diet-dependent in that PACAP actually increases binge feeding in DIO OVX females.

Nevertheless, future research will be imperative to fully evaluate the paradoxical function of PACAP in regulating energy homeostasis not only under normophysiologic conditions and positive energy (i.e., DIO) but also under negative energy states (i.e., food-restriction). In addition, the underlying mechanism of how PACAP exerts its pleiotropic actions not only in the hypothalamic areas and other brain regions, but also in the context of sex differences will need to be further addressed. For example, how might ER- and PAC1 receptor-mediated signaling converge to modulate the ingestion of palatable food in DIO females? What forms of synaptic plasticity might be occurring that would allow projections of VMN PACAP neurons to potentially target other VTA neural substrates in DIO females? Lastly, what are the activational effects of testosterone in males that may contribute to sexually differentiated PAC1 receptor-mediated regulation of energy homeostasis? Only in this way will we unravel the mysteries and further advance our understanding of how PACAP and its receptor adaptively regulate energy balance in response to dynamic changes in energy status.

## Author Contributions

NL, SS, and VM-P performed all metabolic studies. NL performed all electrophysiological recordings. NL and EW created all figures and performed all statistical analyses. NL and EW generated the manuscript, while all authors edited the final manuscript. All authors have read and agreed to the published version of the manuscript.

## Funding

This work was supported by PHS Grant DA024314 and intramural funding from Western University of Health Sciences.

## Conflict of Interest

The authors declare that the research was conducted in the absence of any commercial or financial relationships that could be construed as a potential conflict of interest.

## Publisher’s Note

All claims expressed in this article are solely those of the authors and do not necessarily represent those of their affiliated organizations, or those of the publisher, the editors and the reviewers. Any product that may be evaluated in this article, or claim that may be made by its manufacturer, is not guaranteed or endorsed by the publisher.

## Glossary

**Table:**

α-MSH	Alpha-melanocyte-stimulating hormone
A_10_ DA neurons	Mesolimbic Dopamine neurons
AAV	Adeno-associated virus
AgRP	Agouti-related peptide
AMPA	alpha-amino-3-hydroxyl-5-methyl-4 -isoxazole
AMPK	AMP-activated protein kinase
ARC	Arcuate nucleus
BDNF	Brain-derived neurotrophic factor
BNST	Bed nucleus of stria terminalis
CART	cocaine-and amphetamine-regulated transcript
DIO	Diet-induced obesity
DMN	Dorsomedial nucleus
DREADDs	Designer receptors exclusively activated by designer drugs
GABA	neurotransmitter gamma-aminobutyric acid
GHSR	growth hormone secretagogue receptor
GLP-1	Glucagon like peptide-1
Gq-mER	Gq-coupled membrane estrogen receptor
HFD	High-fat diet
ICV	Intracerebroventricular
IP	Intraperitoneal
IR	Insulin receptor
JAK/STAT	Janus kinase/signal-transducer- and activator-of-transcription
K_ATP_	ATP-gated potassium
LepRb	Leptin receptor
LH	Lateral hypothalamus
MCH	Melanin-concentrating hormone
MC4R	Melanocortin 4 receptor
NAc	Nucleus accumbens
NPY	Neuropeptide Y
NTS	Nucleus tractus solitarius
OVX	Ovariectomized
PACAP	Pituitary Adenylate Cyclase-Activating Polypeptide
PAC1R	PACAP-specific 1 receptor
PI3K	phosphatidylinositol-4,5-bisphosphate 3 kinase
POMC	proopiomelanocortin
PVN	Paraventricular nucleus
SF-1	Steroidogenic factor-1
TRPC5	Transient receptor potential cation 5
VMN	Ventromedial nucleus
VTA	Ventral Tegmental Area
VGlut2	Vesicular glutamate transporters 2

## References

[1] Roh E Kim MS. Brain Regulation of Energy Metabolism. Endocrinol Metab (Seoul) (2016) 31(4):519–24. doi: 10.3803/EnM.2016.31.4.519 PMC5195827 28029023

[2] Travagli RA Anselmi L. Vagal Neurocircuitry and Its Influence on Gastric Motility. Nat Rev Gastroenterol Hepatol (2016) 13(7):389–401. doi: 10.1038/nrgastro.2016.76 27251213 PMC5605144

[3] Schneeberger M Parolari L Das Banerjee T Bhave V Wang P Patel B. Regulation of Energy Expenditure by Brainstem Gaba Neurons. Cell (2019) 178(3):672–85.e12. doi: 10.1016/j.cell.2019.05.048 31257028 PMC7481042

[4] Grijalva CV Novin D. The Role of the Hypothalamus and Dorsal Vagal Complex in Gastrointestinal Function and Pathophysiology. Ann N Y Acad Sci (1990) 597:207–22. doi: 10.1111/j.1749-6632.1990.tb16169.x 2167033

[5] Zheng H Patterson LM Berthoud H-R. Cart in the Dorsal Vagal Complex: Sources of Immunoreactivity and Effects on Fos Expression and Food Intake. Brain Res (2002) 957:298–310. doi: 10.1016/S0006-8993(02)03640-5 12445972

[6] Appleyard SM Bailey TW Doyle MW Jin Y-H Smart JL Low MJ. Proopiomelanocortin Neurons in Nucleus Tractus Solitarius Are Activated by Visceral Afferents: Regulation by Cholecystokinin and Opioids. J Neurosci (2005) 25:3578–85. doi: 10.1523/JNEUROSCI.4177-04.2005 PMC6725389 15814788

[7] Liu J Conde K Zhang P Lilascharoen V Xu Z Lim BK. Enhanced Ampa Receptor Trafficking Mediates the Anorexigenic Effect of Endogenous Glucagon- Like Peptide-1 in the Paraventricular Hypothalamus. Neuron (2017) 96:1–13. doi: 10.2139/ssrn.3155507 29056294 PMC5729931

[8] Ellacott KL Halatchev IG Cone RD. Characterization of Leptin-Responsive Neurons in the Caudal Brainstem. Endocrinology (2006) 147(7):3190–5. doi: 10.1210/en.2005-0877 16601142

[9] Chen J Cheng M Wang L Zhang L Xu D Cao P. A Vagal-Nts Neural Pathway That Stimulates Feeding. Curr Biol (2020) 30(20):3986–98.e5. doi: 10.1016/j.cub.2020.07.084 32822608

[10] Gil K Bugajski A Thor P. Electrical Vagus Nerve Stimulation Decreases Food Consumption and Weight Gain in Rats Fed a High-Fat Diet. J Physiol Pharmacol (2011) 62:637–46. 22314566

[11] Burdyga G Lal S Varro A Dimaline R Thompson DG Dockray GJ. Expression of Cannabinoid Cb1 Receptors by Vagal Afferent Neurons Is Inhibited by Cholecystokinin. J Neurosci (2004) 24:2708–15. doi: 10.1523/JNEUROSCI.5404-03.2004 PMC6729520 15028763

[12] Washington MC Raboin SJ Thompson W Larsen CJ Sayegh AI. Exenatide Reduces Food Intake and Activates the Enteric Nervous System of the Gastrointestinal Tract and the Dorsal Vagal Complex of the Hindbrain in the Rat by a Glp-1 Receptor. Brain Res (2010) 1344:124–33. doi: 10.1016/j.brainres.2010.05.002 20452329

[13] De Silva A Salem V Long CJ Makwana A Newbould RD Rabiner EA. The Gut Hormones Pyy3-36 and Glp-17-36 Amide Reduce Food Intake and Modulate Brain Activity in Appetite Centers in Humans. Cell Metab (2011) 14:700–6. doi: 10.1016/j.cmet.2011.09.010 PMC3267038 22000927

[14] Reimer RA Maurer AD Eller LK Hallam MC Shaykhutdinov R Vogel HJ. Satiety Hormone and Metabolic Response to an Intermittent High Energy Diet Differs in Rats Consuming Long-Term Diets High in Protein or Prebiotic Fiber. J Proteome Res (2012) 11:4065–74. doi: 10.1021/pr300487s PMC3411197 22788871

[15] Cai XJ Evans ML Lister CA Leslie RA Arch JRS Wilson S. Hypoglycemia Activates Orexin Neurons and Selectively Increases Hypothalamic Orexin-B Levels: Responses Inhibited by Feeding and Possibly Mediated by the Nucleus of the Solitary Tract. Diabetes (2001) 50:105–12. doi: 10.2337/diabetes.50.1.105 11147774

[16] Ciriello J McMurray JC Babic T de Oliveira CV. Collateral Axonal Projections From Hypothalamic Hypocretin Neurons to Cardiovascular Sites in Nucleus Ambiguus and Nucleus Tractus Solitarius. Brain Res (2003) 991(1-2):133–41. doi: 10.1016/j.brainres.2003.08.016 14575885

[17] Gasparini S Howland JM Thatcher AJ Geerling JC. Central Afferents to the Nucleus of the Solitary Tract in Rats and Mice. J Comp Neurol (2020) 528(16):2708–28. doi: 10.1002/cne.24927 PMC7942812 32307700

[18] Zheng H Patterson LM Berthoud HR. Orexin-A Projections to the Caudal Medulla and Orexin-Induced C-Fos Expression, Food Intake, and Autonomic Function. J Comp Neurol (2005) 485(2):127–42. doi: 10.1002/cne.20515 15776447

[19] Parise EM Lilly N Kay K Dossat AM Seth R Overton JM. Evidence for the Role of Hindbrain Orexin-1 Receptors in the Control of Meal Size. Am J Physiol Regul Integr Comp Physiol (2011) 301(6):R1692–9. doi: 10.1152/ajpregu.00044.2011 PMC3233858 21957165

[20] Anand BK Brobeck JR. Hypothalamic Control of Food Intake in Rats and Cats. Yale J Biol Med (1951) 24(2):123–40. PMC2599116 14901884

[21] Olney JW. Brain Lesions, Obesity, and Other Disturbances in Mice Treated With Monosodium Glutamate. Science (1969) 164:719–21. doi: 10.1126/science.164.3880.719 5778021

[22] Leibowitz SF Hammer NJ Chang K. Hypothalamic Paraventricular Nucleus Lesions Produce Overeating and Obesity in the Rat. Physiol Behav (1981) 27(6):1031–40. doi: 10.1016/0031-9384(81)90366-8 7335803

[23] Cowley MA Cone RD Enriori P Louiselle I Williams SM Evans AE. Electrophysiological Actions of Peripheral Hormones on Melanocortin Neurons. Ann NY Acad Sci (2003) 994:175–86. doi: 10.1111/j.1749-6632.2003.tb03178.x 12851314

[24] Hill JW Williams KW Ye C Luo J Balthasar N Coppari R. Acute Effects of Leptin Require Pi3k Signaling in Hypothalamic Proopiomelanocortin Neurons in Mice. J Clin Invest (2008) 118:1796–805. doi: 10.1172/JCI32964 PMC2276395 18382766

[25] Qiu J Fang Y Rønnekleiv OK Kelly MJ. Leptin Excites Proopiomelanocortin Neurons *via* Activation of Trpc Channels. J Neurosci (2010) 30:1560–5. doi: 10.1523/JNEUROSCI.4816-09.2010 PMC3095824 20107083

[26] van den Top M Lee K Whyment AD Blanks AM Spanswick D. Orexigen-Sensitive Npy/Agrp Pacemaker Neurons in the Hypothalamic Arcuate Nucleus. Nat Neurosci (2004) 5:493–4. doi: 10.1038/nn1226 15097991

[27] Baver SB Hope K Guyot S Bjørbaek C Kaczorowski C O'Connell KM. Leptin Modulates the Intrinsic Excitability of Agrp/Npy Neurons in the Arcuate Nucleus of the Hypothalamus. J Neurosci (2014) 34(16):5486–96. doi: 10.1523/jneurosci.4861-12.2014 PMC4298648 24741039

[28] Klöckener T Hess S Belgardt BF Paeger L Verhagen LAW Husch A. High-Fat Feeding Promotes Obesity *via* Insulin Receptor/Pi3k-Dependent Inhibition of Sf-1 Vmh Neurons. Nat Neurosci (2011) 14:911–8. doi: 10.1038/nn.2847 PMC3371271 21642975

[29] Qiu J Zhang C Borgquist A Nestor CC Smith AW Bosch MA. Insulin Excites Anorexigenic Proopiomelanocortin Neurons *via* Activation of Canonical Transient Receptor Potential Channels. Cell Metab (2014) 19:682–93. doi: 10.1016/j.cmet.2014.03.004 PMC4183666 24703699

[30] Huang Y He Z Gao Y Lieu L Yao T Sun J. Phosphoinositide 3-Kinase Is Integral for the Acute Activity of Leptin and Insulin in Male Arcuate Npy/Agrp Neurons. J Endocr Soc (2018) 2(6):518–32. doi: 10.1210/js.2018-00061 PMC5961025 29850651

[31] Qiu J Wagner EJ Ronnekleiv OK Kelly MJ. Insulin and Leptin Excite Anorexigenic Pro-Opiomelanocortin Neurones *via* Activation of Trpc5 Channels. J Neuroendocrinol (2017) 30(2). doi: 10.1111/jne.12501 PMC5957276 28675783

[32] Plum L Ma X Hampel B Balthasar N Coppari R Münzberg H. Enhanced Pip_3_ Signaling in Pomc Neurons Causes Katp Channel Activation and Leads to Diet-Sensitive Obesity. J Clin Invest (2006) 116:1886–901. doi: 10.1172/JCI27123 PMC1481658 16794735

[33] Williams KW Margatho LO Lee CE Choi M Lee S Scott MM. Segregation of Acute Leptin and Insulin Effects in Distinct Populations of Arcuate Proopiomelanocortin Neurons. J Neurosci (2010) 30:2472–9. doi: 10.1523/JNEUROSCI.3118-09.2010 PMC2836776 20164331

[34] Dodd GT Michael NJ Lee-Young RS Mangiafico SP Pryor JT Munder AC. Insulin Regulates Pomc Neuronal Plasticity to Control Glucose Metabolism. eLife (2018) 7:e38704. doi: 10.7554/eLife.38704 30230471 PMC6170188

[35] Chen HY Trumbauer ME Chen AS Weingarth DT Adams JR Frazier EG. Orexigenic Action of Peripheral Ghrelin Is Mediated by Neuropeptide Y and Agouti-Related Protein. Endocrinology (2004) 145(6):2607–12. doi: 10.1210/en.2003-1596 14962995

[36] Chen SR Chen H Zhou JJ Pradhan G Sun Y Pan HL. Ghrelin Receptors Mediate Ghrelin-Induced Excitation of Agouti-Related Protein/Neuropeptide Y But Not Pro-Opiomelanocortin Neurons. J Neurochem (2017) 142(4):512–20. doi: 10.1111/jnc.14080 28547758

[37] Kalivas PW Duffy P. Sensitization to Repeated Morphine Injection in the Rat: Possible Involvement of A_10_ Dopamine Neurons. J Pharmacol Exp Ther (1987) 241:204–12. 3572784

[38] Wise RA. Role of Brain Dopamine in Food Reward and Reinforcement. Phil Trans R Soc B (2006) 361:1149–58. doi: 10.1098/rstb.2006.1854 PMC1642703 16874930

[39] Durst M Konczol K Balazsa T Eyre MD Toth ZE. Reward-Representing D1-Type Neurons in the Medial Shell of the Accumbens Nucleus Regulate Palatable Food Intake. Int J Obes (2019) 43(4):917–27. doi: 10.1038/s41366-018-0133-y PMC6484714 29907842

[40] Bingham NC Anderson KK Reuter AL Stallings NR Parker KL. Selective Loss of Leptin Receptors in the Ventromedial Hypothalamic Nucleus Results in Increased Adiposity and a Metabolic Syndrome. Endocrinology (2008) 149(5):2138–48. doi: 10.1210/en.2007-1200 PMC2329259 18258679

[41] Zhang R Dhillon H Yin H Yoshimura A Lowell BB Maratos-Flier E. Selective Inactivation of Socs3 in Sf1 Neurons Improves Glucose Homeostasis Without Affecting Body Weight. Endocrinology (2008) 149(11):5654–61. doi: 10.1210/en.2008-0805 PMC2584592 18669597

[42] Kim KW Zhao L Donato J Jr. Kohno D Xu Y Elias CF. Steroidogenic Factor 1 Directs Programs Regulating Diet-Induced Thermogenesis and Leptin Action in the Ventral Medial Hypothalamic Nucleus. Proc Natl Acad Sci USA (2011) 108(26):10673–8. doi: 10.1073/pnas.1102364108 PMC3127910 21636788

[43] Majdic G Young M Gomez-Sanchez E Anderson P Szczepaniak LS Dobbins RL. Knockout Mice Lacking Steroidogenic Factor 1 Are a Novel Genetic Model of Hypothalamic Obesity. Endocrinology (2002) 143:607–14. doi: 10.1210/endo.143.2.8652 11796516

[44] Dhillon H Zigman JM Ye C Lee CE McGovern RA Tang V. Leptin Directly Activates Sf1 Neurons in the Vmh, and This Action by Leptin Is Required for Normal Body-Weight Homeostasis. Neuron (2006) 49:191–203. doi: 10.1016/j.neuron.2005.12.021 16423694

[45] Lyons WE Mamounas LA Ricaurte GA Coppola V Reid SW Bora SH. Brain-Derived Neurotrophic Factor-Deficient Mice Develop Aggressiveness and Hyperphagia in Conjunction With Brain Serotonergic Abnormalities. Proc Natl Acad Sci USA (1999) 96(26):15239–44. doi: 10.1073/pnas.96.26.15239 PMC24804 10611369

[46] Yeo GS Connie Hung CC Rochford J Keogh J Gray J Sivaramakrishnan S. A *De Novo* Mutation Affecting Human Trkb Associated With Severe Obesity and Developmental Delay. Nat Neurosci (2004) 7(11):1187–9. doi: 10.1038/nn1336 15494731

[47] Xu B Goulding EH Zang K Cepoi D Cone RD Jones KR. Brain-Derived Neurotrophic Factor Regulates Energy Balance Downstream of Melanocortin-4 Receptor. Nat Neurosci (2003) 6(7):736–42. doi: 10.1038/nn1073 PMC2710100 12796784

[48] Sternson SM Shepherd GMG Friedman JM. Topographic Mapping of Vmh-Arcuate Nucleus Microcirsuits and Their Reoganization by Fasting. Nat Neurosci (2005) 8:1356–63. doi: 10.1038/nn1550 16172601

[49] Fabelo C Hernandez J Chang R Seng S Alicea N Tian S. Endocannabinoid Signaling at Hypothalamic Steroidogenic Factor-1/Proopiomelanocortin Synapses Is Sex- and Diet-Sensitive. Front Mol Neurosci (2018) 11:214. doi: 10.3389/fnmol.2018.00214 29973869 PMC6020785

[50] Chang R Hernandez J Gastelum C Guadagno K Perez L Wagner EJ. Pituitary Adenylate Cyclase-Activating Polypeptide Excites Proopiomelanocortin Neurons: Implications for the Regulation of Energy Homeostasis. Neuroendocrinology (2021) 111:45–69. doi: 10.1159/000506367 32028278

[51] Glembotski CC. Subcellular Fractionation Studies on the Post-Translational Processing of Pro-Adrenocorticotropic Hormone/Endorphin in Rat Intermediate Pituitary. J Biol Chem (1981) 256(14):7433–9. doi: 10.1016/S0021-9258(19)68981-7 6265449

[52] Glembotski CC. Acetylation of Alpha-Melanotropin and Beta-Endorphin in the Rat Intermediate Pituitary. Subcellular Localization. J Biol Chem (1982) 257(17):10493–500. doi: 10.1016/S0021-9258(18)34045-6 6286656

[53] Yaswen L Diehl N Brennan MB Hochgeschwender U. Obesity in the Mouse Model of Pro-Opiomelanocortin Deficiency Responds to Peripheral Melanocortin. Nat Med (1999) 5(9):1066–70. doi: 10.1038/12506 10470087

[54] Broberger C Johansen J Johansson C Schalling M Hökfelt T. The Neuropeptide Y/Agouti-Related Protein (Agrp) Brain Circuitry in Normal, Anoretic, and Monosodium Glutamate-Treated Mice. Proc Natl Acad Sci (1998) 95:15043–8. doi: 10.1073/pnas.95.25.15043 PMC24572 9844012

[55] Fan W Boston BA Kesterson RA Hruby VJ Cone RD. Role of Melanocortinergic Neurons in Feeding and the Agouti Obesity Syndrome. Nature (1997) 385:165–8. doi: 10.1038/385165a0 8990120

[56] Belgardt BF Okamura T Brüning JC. Hormone and Glucose Signalling in Pomc and Agrp Neurons. J Physiol (Lond ) (2009) 587:5305–14. doi: 10.1113/jphysiol.2009.179192 PMC2793863 19770186

[57] Poggioli R Vergoni AV Bertolini A. Acth-(1-24) and Alpha-Msh Antagonize Feeding Behavior Stimulated by Kappa Opiate Agonists. Peptides (1986) 7(5):843–8. doi: 10.1016/0196-9781(86)90104-x 3025825

[58] Huszar D Lynch CA Fairchild-Huntress V Dunmore JH Fang Q Berkemeier LR. Targeted Disruption of the Melanocortin-4 Receptor Results in Obesity in Mice. Cell (1997) 88:131–41. doi: 10.1016/S0092-8674(00)81865-6 9019399

[59] Kelly MJ Loose MD Ronnekleiv OK. Opioids Hyperpolarize β-Endorphin Neurons *via* μ-Receptor Activation of a Potassium Conductance. Neuroendocrinology (1990) 52:268–75. doi: 10.1159/000125597 2170854

[60] Loose MD Kelly MJ. Opioids Act at μ-Receptors to Hyperpolarize Arcuate Neurons *via* an Inwardly Rectifying Potassium Conductance. Brain Res (1990) 513:15–23. doi: 10.1016/0006-8993(90)91084-T 2161696

[61] Loose MD Ronnekleiv OK Kelly MJ. Neurons in the Rat Arcuate Nucleus are Hyperpolarized by Gaba(B) and μ-Opioid Receptor Agonists: Evidence for Convergence at a Ligand-Gated Potassium Conductance. Neuroendocrinology (1991) 54:537–44. doi: 10.1159/000125979 1664497

[62] Pennock RL Hentges ST. Differential Expression and Sensitivity of Presynaptic and Postsynaptic Opioid Receptors Regulating Hypothalamic Proopiomelanocortin Neurons. J Neurosci (2011) 31(1):281–8. doi: 10.1523/jneurosci.4654-10.2011 PMC3061472 21209213

[63] Grandison L Guidotti A. Stimulation of Food Intake by Muscimol and Beta Endorphin. Neuropharmacology (1977) 16(7-8):533–6. doi: 10.1016/0028-3908(77)90019-3 917261

[64] Kalra SP Horvath TL. Neuroendocrine Interactions Between Galanin, Opioids and Neuropeptide Y in the Control of Reproduction and Appetite. Ann NY Acad Sci (1998) 863:236–40. doi: 10.1111/j.1749-6632.1998.tb10698.x 9928174

[65] Dutia R Meece K Dighe S Kim AJ Wardlaw SL. B-Endorphin Antagonizes the Effects of A-Msh on Food Intake and Body Weight. Endocrinology (2012) 153(9):4246–55. doi: 10.1210/en.2012-1166 PMC3423622 22778225

[66] Appleyard SM Hayward M Young JI Butler AA Cone RD Rubinstein M. A Role for Endogenous β-Endorphin in Energy Homeostasis. Endocrinology (2003) 144:1753–60. doi: 10.1210/en.2002-221096 12697680

[67] Elias CF Lee C Kelly J Aschkenasi C Ahima RS Couceyro PR. Leptin Activates Hypothalamic Cart Neurons Projecting to the Spinal Cord. Neuron (1998) 21(6):1375–85. doi: 10.1016/s0896-6273(00)80656-x 9883730

[68] Tong Q Ye C-P Jones JE Elmquist JK Lowell BB. Synaptic Release of Gaba by Agrp Neurons Is Required for Normal Regulation of Energy Balance. Nat Neurosci (2008) 11:998–1000. doi: 10.1038/nn.2167 19160495 PMC2662585

[69] Nguyen AD Mitchell NF Lin S Macia L Yulyaningsih E Baldock PA. Y1 and Y5 Receptors Are Both Required for the Regulation of Food Intake and Energy Homeostasis in Mice. PloS One (2012) 7(6):e40191. doi: 10.1371/journal.pone.0040191 22768253 PMC3387009

[70] Sohn JW Elmquist JK Williams KW. Neuronal Circuits That Regulate Feeding Behavior and Metabolism. Trends Neurosci (2013) 36(9):504–12. doi: 10.1016/j.tins.2013.05.003 PMC3769497 23790727

[71] Clark JT Kalra PS Crowley WR Kalra SP. Neuropeptide Y and Human Pancreatic Polypeptide Stimulate Feeding Behavior in Rats. Endocrinology (1984) 115(1):427–9. doi: 10.1210/endo-115-1-427 6547387

[72] Morley JE Levine AS Gosnell BA Kneip J Grace M. Effect of Neuropeptide Y on Ingestive Behaviors in the Rat. Am J Physiol (1987) 252(3 Pt 2):R599–609. doi: 10.1152/ajpregu.1987.252.3.R599 3826420

[73] Leibowitz SF Sladek C Spencer L Tempel D. Neuropeptide Y, Epinephrine and Norepinephrine in the Paraventricular Nucleus: Stimulation of Feeding and the Release of Corticosterone, Vasopressin and Glucose. Brain Res Bull (1988) 21(6):905–12. doi: 10.1016/0361-9230(88)90025-1 3224284

[74] Stanley BG Leibowitz SF. Neuropeptide Y Injected in the Paraventricular Hypothalamus: A Powerful Stimulant of Feeding Behavior. Proc Natl Acad Sci USA (1985) 82(11):3940–3. doi: 10.1073/pnas.82.11.3940 PMC397905 3858854

[75] Flood JF Morley JE. Increased Food Intake by Neuropeptide Y Is Due to an Increased Motivation to Eat. Peptides (1991) 12(6):1329–32. doi: 10.1016/0196-9781(91)90215-b 1815219

[76] Niimi M Sato M Taminato T. Neuropeptide Y in Central Control of Feeding and Interactions With Orexin and Leptin. Endocrine (2001) 14(2):269–73. doi: 10.1385/endo:14:2:269 11394646

[77] Graham M Shutter JR Sarmiento U Sarosi I Stark KL. Overexpression of Agrt Leads to Obesity in Transgenic Mice. Nat Genet (1997) 17(3):273–4. doi: 10.1038/ng1197-273 9354787

[78] Ollmann MM Wilson BD Yang YK Kerns JA Chen Y Gantz I. Antagonism of Central Melanocortin Receptors *In Vitro* and *In Vivo* by Agouti-Related Protein. Science (1997) 278(5335):135–8. doi: 10.1126/science.278.5335.135 9311920

[79] Small CJ Liu YL Stanley SA Connoley IP Kennedy A Stock MJ. Chronic Cns Administration of Agouti-Related Protein (Agrp) Reduces Energy Expenditure. Int J Obes Relat Metab Disord (2003) 27(4):530–3. doi: 10.1038/sj.ijo.0802253 12664087

[80] Krashes MJ Koda S Ye C Rogan SC Adams AC Cusher DS. Rapid, Reversible Activation of Agrp Neurons Drives Feeding Behavior in Mice. J Clin Invest (2011) 121:1424–8. doi: 10.1172/JCI46229 PMC3069789 21364278

[81] Krashes MJ Shah BP Koda S Lowell BB. Rapid Versus Delayed Stimulation of Feeding by the Endogenously Released Agrp Neuron Mediators Gaba, Npy, and Agrp. Cell Metab (2013) 18(4):588–95. doi: 10.1016/j.cmet.2013.09.009 PMC3822903 24093681

[82] Palmiter RD Erickson JC Hollopeter G Baraban SC Schwartz MW. Life Without Neuropeptide Y. Recent Prog Horm Res (1998) 53:163–99. 9769708

[83] Qian S Chen H Weingarth D Trumbauer ME Novi DE Guan X. Neither Agouti-Related Protein Nor Neuropeptide Y Is Critically Required for the Regulation of Energy Homeostasis in Mice. Mol Cell Biol (2002) 22(14):5027–35. doi: 10.1128/mcb.22.14.5027-5035.2002 PMC139785 12077332

[84] Corander MP Rimmington D Challis BG O'Rahilly S Coll AP. Loss of Agouti-Related Peptide Does Not Significantly Impact the Phenotype of Murine Pomc Deficiency. Endocrinology (2011) 152(5):1819–28. doi: 10.1210/en.2010-1450 PMC3137464 21363936

[85] Luquet S Perez FA Hnasko TS Palmiter RD. Npy/Agrp Neurons are Essential for Feeding in Adult Mice But Can Be Ablated in Neonates. Science (2005) 310(5748):683–5. doi: 10.1126/science.1115524 16254186

[86] Jais A Paeger L Sotelo-Hitschfeld T Bremser S Prinzensteiner M Klemm P. Pnoc^arc^ Neurons Promote Hyperphagia and Obesity Upon High-Fat Feeding. Neuron (2020) 106:1–17. doi: 10.1016/j.neuron.2020.03.022 32302532 PMC7303947

[87] Hernandez J Perez L Soto R Le N Gastelum C Wagner EJ. Nociceptin/Orphanin Fq Neurons in the Arcuate Nucleus and Ventral Tegmental Area Act *via* Nociceptin Opioid Peptide Receptor Signaling to Inhibit Proopiomelanocortin and a 10 Dopamine Neurons and Thereby Modulate Ingestion of Palatable Food. Physiol Behav (2021) 228:113183. doi: 10.1016/j.physbeh.2020.113183 32979341 PMC7736116

[88] Stuber GD Wise RA. Lateral Hypothalamic Circuits for Feeding and Reward. Nat Neurosci (2016) 19(2):198–205. doi: 10.1038/nn.4220 26814589 PMC4927193

[89] Qu D Ludwig DS Gammeltoft S Piper M Pelleymounter MA Cullen MJ. A Role for Melanin-Concentrating Hormone in the Central Regulation of Feeding Behaviour. Nature (1996) 380(6571):243–7. doi: 10.1038/380243a0 8637571

[90] Rossi M Choi SJ O'Shea D Miyoshi T Ghatei MA Bloom SR. Melanin-Concentrating Hormone Acutely Stimulates Feeding, But Chronic Administration Has No Effect on Body Weight. Endocrinology (1997) 138(1):351–5. doi: 10.1210/endo.138.1.4887 8977423

[91] Ludwig DS Tritos NA Mastaitis JW Kulkarni R Kokkotou E Elmquist J. Melanin-Concentrating Hormone Overexpression in Transgenic Mice Leads to Obesity and Insulin Resistance. J Clin Invest (2001) 107(3):379–86. doi: 10.1172/jci10660 PMC199192 11160162

[92] Della-Zuana O Presse F Ortola C Duhault J Nahon JL Levens N. Acute and Chronic Administration of Melanin-Concentrating Hormone Enhances Food Intake and Body Weight in Wistar and Sprague-Dawley Rats. Int J Obes (2002) 26:1289–95. doi: 10.1038/sj.ijo.0802079 12355323

[93] Shimada M Tritos NA Lowell BB Flier JS Maratos-Flier E. Mice Lacking Melanin-Concentrating Hormone are Hypophagic and Lean. Nature (1998) 396(6712):670–4. doi: 10.1038/25341 9872314

[94] Marsh DJ Weingarth DT Novi DE Chen HY Trumbauer ME Chen AS. Melanin-Concentrating Hormone 1 Receptor-Deficient Mice Are Lean, Hyperactive, and Hyperphagic and Have Altered Metabolism. Proc Natl Acad Sci USA (2002) 99(5):3240–5. doi: 10.1073/pnas.052706899 PMC122503 11867747

[95] Sakurai T Amemiya A Ishii M Matsuzaki I Chemelli RM Tanaka H. Orexins and Orexin Receptors: A Family of Hypothalamic Neuropeptides and G Protein-Coupled Receptors That Regulate Feeding Behavior. Cell (1998) 92(4):573–85. doi: 10.1016/s0092-8674(00)80949-6 9491897

[96] Zink AN Bunney PE Holm AA Billington CJ Kotz CM. Neuromodulation of Orexin Neurons Reduces Diet-Induced Adiposity. Int J Obes (Lond) (2018) 42(4):737–45. doi: 10.1038/ijo.2017.276 PMC5962366 29180723

[97] Dube MG Kalra SP Kalra PS. Food Intake Elicited by Central Administration of Orexins/Hypocretins: Identification of Hypothalamic Sites of Action. Brain Res (1999) 842(2):473–7. doi: 10.1016/s0006-8993(99)01824-7 10526145

[98] Cason AM Smith RJ Tahsili-Fahadan P Moorman DE Sartor GC Aston-Jones G. Role of Orexin/Hypocretin in Reward-Seeking and Addiction: Implications for Obesity. Physiol Behav (2010) 100(5):419–28. doi: 10.1016/j.physbeh.2010.03.009 PMC2886173 20338186

[99] Ma X Zubcevic L Brüning JC Ashcroft FM Burdakov D. Electrical Inhibition of Identified Anorexigenic Pomc Neurons by Orexin/Hypocretin. J Neurosci (2007) 27:1529–33. doi: 10.1523/JNEUROSCI.3583-06.2007 PMC6673748 17301161

[100] Jo Y-H Chen Y-JL Chua SC Talmage DA Role LW. Integration of Endocannabinoid and Leptin Signaling in an Appetite-Related Neural Circuit. Neuron (2005) 48:1055–66. doi: 10.1016/j.neuron.2005.10.021 PMC2280039 16364907

[101] Fekete C Légrádi G Mihály E Huang QH Tatro JB Rand WM. Alpha-Melanocyte-Stimulating Hormone is Contained in Nerve Terminals Innervating Thyrotropin-Releasing Hormone-Synthesizing Neurons in the Hypothalamic Paraventricular Nucleus and Prevents Fasting-Induced Suppression of Prothyrotropin-Releasing Hormone Gene Expression. J Neurosci (2000) 20(4):1550–8. doi: 10.1523/jneurosci.20-04-01550.2000 PMC6772359 10662844

[102] Kim MS Rossi M Abusnana S Sunter D Morgan DG Small CJ. Hypothalamic Localization of the Feeding Effect of Agouti-Related Peptide and Alpha-Melanocyte-Stimulating Hormone. Diabetes (2000) 49(2):177–82. doi: 10.2337/diabetes.49.2.177 10868932

[103] Bi S Ladenheim EE Schwartz GJ Moran TH. A Role for Npy Overexpression in the Dorsomedial Hypothalamus in Hyperphagia and Obesity of Oletf Rats. Am J Physiol Regul Integr Comp Physiol (2001) 281(1):R254–60. doi: 10.1152/ajpregu.2001.281.1.R254 11404301

[104] Chao PT Yang L Aja S Moran TH Bi S. Knockdown of Npy Expression in the Dorsomedial Hypothalamus Promotes Development of Brown Adipocytes and Prevents Diet-Induced Obesity. Cell Metab (2011) 13(5):573–83. doi: 10.1016/j.cmet.2011.02.019 PMC3093161 21531339

[105] Liu H Kishi T Roseberry AG Cai X Lee CE Montez JM. Transgenic Mice Expressing Green Fluorescent Protein Under the Control of the Melanocortin-4 Receptor Promoter. J Neurosci (2003) 23(18):7143–54. doi: 10.1523/jneurosci.23-18-07143.2003 PMC6740648 12904474

[106] Tao YX. The Melanocortin-4 Receptor: Physiology, Pharmacology, and Pathophysiology. Endocr Rev (2010) 31(4):506–43. doi: 10.1210/er.2009-0037 PMC3365848 20190196

[107] Beck B Jhanwar-Uniyal M Burlet A Chapleur-Chateau M Leibowitz SF Burlet C. Rapid and Localized Alterations of Neuropeptide Y in Discrete Hypothalamic Nuclei With Feeding Status. Brain Res (1990) 528(2):245–9. doi: 10.1016/0006-8993(90)91664-3 2271925

[108] Shibasaki T Oda T Imaki T Ling N Demura H. Injection of Anti-Neuropeptide Y Gamma-Globulin Into the Hypothalamic Paraventricular Nucleus Decreases Food Intake in Rats. Brain Res (1993) 601(1-2):313–6. doi: 10.1016/0006-8993(93)91727-a 7679310

[109] Guan XM Yu H Trumbauer M Frazier E van der Ploeg LH Chen H. Induction of Neuropeptide Y Expression in Dorsomedial Hypothalamus of Diet-Induced Obese Mice. Neuroreport (1998) 9(15):3415–9. doi: 10.1097/00001756-199810260-00015 9855291

[110] Zhang Y Proenca R Maffei M Barone M Leopold L Friedman JM. Positional Cloning of the Mouse Obese Gene and Its Human Homologue. Nature (1994) 372(6505):425–32. doi: 10.1038/372425a0 7984236

[111] Considine RV Sinha MK Heiman ML Kriauciunas A Stephens TW Nyce MR. Serum Immunoreactive-Leptin Concentrations in Normal-Weight and Obese Humans. N Engl J Med (1996) 334(5):292–5. doi: 10.1056/nejm199602013340503 8532024

[112] Zigman JM Elmquist JK. Minireview: From Anorexia to Obesity–the Yin and Yang of Body Weight Control. Endocrinology (2003) 144:3749–56. doi: 10.1210/en.2003-0241 12933644

[113] Tartaglia LA Dembski M Weng X Deng N Culpepper J Devos R. Identification and Expression Cloning of a Leptin Receptor, Ob-R. Cell (1995) 83(7):1263–71. doi: 10.1016/0092-8674(95)90151-5 8548812

[114] Chen H Charlat O Tartaglia LA Woolf EA Weng X Ellis SJ. Evidence That the Diabetes Gene Encodes the Leptin Receptor: Identification of a Mutation in the Leptin Receptor Gene in Db/Db Mice. Cell (1996) 84(3):491–5. doi: 10.1016/s0092-8674(00)81294-5 8608603

[115] Lee GH Proenca R Montez JM Carroll KM Darvishzadeh JG Lee JI. Abnormal Splicing of the Leptin Receptor in Diabetic Mice. Nature (1996) 379(6566):632–5. doi: 10.1038/379632a0 8628397

[116] Schwartz MW Baskin DG Bukowski TR Kuijper JL Foster D Lasser G. Specificity of Leptin Action on Elevated Blood Glucose Levels and Hypothalamic Neuropeptide Y Gene Expression in Ob/Ob Mice. Diabetes (1996) 45(4):531–5. doi: 10.2337/diab.45.4.531 8603777

[117] Pinto S Roseberry AG Liu H Diano S Shanabrough M Cai X. Rapid Rewiring of Arcuate Nucleus Feeding Circuits by Leptin. Science (2004) 304:110–5. doi: 10.1126/science.1089459 15064421

[118] Halaas JL Gajiwala KS Maffei M Cohen SL Chait BT Rabinowitz D. Weight-Reducing Effects of the Plasma Protein Encoded by the Obese Gene. Science (1995) 269(5223):543–6. doi: 10.1126/science.7624777 7624777

[119] Montague CT Farooqi IS Whitehead JP Soos MA Rau H Wareham NJ. Congenital Leptin Deficiency Is Associated With Severe Early-Onset Obesity in Humans. Nature (1997) 387(6636):903–8. doi: 10.1038/43185 9202122

[120] Clément K Vaisse C Lahlou N Cabrol S Pelloux V Cassuto D. A Mutation in the Human Leptin Receptor Gene Causes Obesity and Pituitary Dysfunction. Nature (1998) 392:398–401. doi: 10.1038/32911 9537324

[121] Havrankova J Roth J Brownstein M. Insulin Receptors Are Widely Distributed in the Central Nervous System of the Rat. Nature (1978) 272(5656):827–9. doi: 10.1038/272827a0 205798

[122] Corp ES Woods SC Porte D Jr. Dorsa DM Figlewicz DP Baskin DG. Localization of 125i-Insulin Binding Sites in the Rat Hypothalamus by Quantitative Autoradiography. Neurosci Lett (1986) 70(1):17–22. doi: 10.1016/0304-3940(86)90430-1 3534636

[123] Benoit SC Air EL Coolen LM Strauss R Jackman A Clegg DJ. The Catabolic Action of Insulin in the Brain Is Mediated by Melanocortins. J Neurosci (2002) 22(20):9048–52. doi: 10.1523/jneurosci.22-20-09048.2002 PMC6757684 12388611

[124] Könner AC Janoschek R Plum L Jordan SD Rother E Ma X. Insulin Action in Agrp-Expressing Neurons Is Required for Suppression of Hepatic Glucose Production. Cell Metab (2007) 5(6):438–49. doi: 10.1016/j.cmet.2007.05.004 17550779

[125] Hill JW Elias CF Fukuda M Williams KW Berglund ED Holland WL. Direct Insulin and Leptin Action on Pro-Opiomelanocortin Neurons Is Required for Normal Glucose Homeostasis and Fertility. Cell Metab (2010) 11:286–97. doi: 10.1016/j.cmet.2010.03.002 PMC2854520 20374961

[126] Shin AC Filatova N Lindtner C Chi T Degann S Oberlin D. Insulin Receptor Signaling in Pomc, But Not Agrp, Neurons Controls Adipose Tissue Insulin Action. Diabetes (2017) 66(6):1560–71. doi: 10.2337/db16-1238 PMC5440019 28385803

[127] Qiu J Bosch MA Zhang C Ronnekleiv OK Kelly MJ. Estradiol Protects Neuropeptide Y/Agouti-Related Peptide Neurons Against Insulin Resistance in Females. Neuroendocrinology (2020) 110:105–18. doi: 10.1159/000501560 PMC6920578 31212279

[128] Qiu J Bosch MA Meza C Navarro U-V Nestor CC Wagner EJ. Estradiol Protects Proopiomelanocortin Neurons Against Insulin Resistance. Endocrinology (2018) 159:647–64. doi: 10.1210/en.2017-00793 PMC5774249 29165691

[129] Dodd GT Decherf S Loh K Simonds SE Wiede F Balland E. Leptin and Insulin Act on Pomc Neurons to Promote the Browning of White Fat. Cell (2015) 160:88–104. doi: 10.1016/j.cell.2014.12.022 25594176 PMC4453004

[130] Dodd GT Andrews ZB Simonds SE Michael NJ DeVeer M Bruning JC. A Hypothalamic Phosphatase Switch Coordinates Energy Expenditure With Feeding. Cell Metab (2017) 26:375–93. doi: 10.1016/j.cmet.2017.07.013 28768176

[131] Dodd GT Xirouchaki CE Eramo M Mitchell CA Andrews ZB Henry BA. Intranasal Targeting of Hypothalamic Ptp1b and Tcptp Reinstates Leptin and Insulin Sensitivity and Promotes Weight Loss in Obesity. Cell Rep (2019) 28:2905–22. doi: 10.1016/j.celrep.2019.08.019 31509751

[132] Schaeffer M Langlet F Lafont C Molino F Hodson DJ Roux T. Rapid Sensing of Circulating Ghrelin by Hypothalamic Appetite-Modifying Neurons. Proc Natl Acad Sci USA (2013) 110(4):1512–7. doi: 10.1073/pnas.1212137110 PMC3557016 23297228

[133] Wang L Saint-Pierre DH Taché Y. Peripheral Ghrelin Selectively Increases Fos Expression in Neuropeptide Y - Synthesizing Neurons in Mouse Hypothalamic Arcuate Nucleus. Neurosci Lett (2002) 325(1):47–51. doi: 10.1016/s0304-3940(02)00241-0 12023064

[134] Tamura H Kamegai J Shimizu T Ishii S Sugihara H Oikawa S. Ghrelin Stimulates Gh But Not Food Intake in Arcuate Nucleus Ablated Rats. Endocrinology (2002) 143(9):3268–75. doi: 10.1210/en.2002-220268 12193538

[135] Riediger T Traebert M Schmid HA Scheel C Lutz TA Scharrer E. Site-Specific Effects of Ghrelin on the Neuronal Activity in the Hypothalamic Arcuate Nucleus. Neurosci Lett (2003) 341(2):151–5. doi: 10.1016/s0304-3940(02)01381-2 12686388

[136] Luquet S Phillips CT Palmiter RD. Npy/Agrp Neurons Are Not Essential for Feeding Responses to Glucoprivation. Peptides (2007) 28(2):214–25. doi: 10.1016/j.peptides.2006.08.036 17194499

[137] Kamegai J Tamura H Shimizu T Ishii S Sugihara H Wakabayashi I. Chronic Central Infusion of Ghrelin Increases Hypothalamic Neuropeptide Y and Agouti-Related Protein Mrna Levels and Body Weight in Rats. Diabetes (2001) 50(11):2438–43. doi: 10.2337/diabetes.50.11.2438 11679419

[138] Nakazato M Murakami N Date Y Kojima M Matsuo H Kangawa K. A Role for Ghrelin in the Central Regulation of Feeding. Nature (2001) 409(6817):194–8. doi: 10.1038/35051587 11196643

[139] Hashiguchi H Sheng Z Routh V Gerzanich V Simard JM Bryan J. Direct Versus Indirect Actions of Ghrelin on Hypothalamic Npy Neurons. PloS One (2017) 12(9):e0184261. doi: 10.1371/journal.pone.0184261 28877214 PMC5587286

[140] Cowley MA Smith RG Diano S Tschöp M Pronchuk N Grove KL. The Distribution and Mechanism of Action of Ghrelin in the Cns Demonstrates a Novel Hypothalamic Circuit Regulating Energy Homeostasis. Neuron (2003) 37(4):649–61. doi: 10.1016/s0896-6273(03)00063-1 12597862

[141] Currie PJ Mirza A Fuld R Park D Vasselli JR. Ghrelin is an Orexigenic and Metabolic Signaling Peptide in the Arcuate and Paraventricular Nuclei. Am J Physiol Regul Integr Comp Physiol (2005) 289(2):R353–r8. doi: 10.1152/ajpregu.00756.2004 15817841

[142] Tschöp M Smiley DL Heiman ML. Ghrelin Induces Adiposity in Rodents. Nature (2000) 407(6806):908–13. doi: 10.1038/35038090 11057670

[143] Yang Y Atasoy D Su HH Sternson SM. Hunger States Switch a Flip-Flop Memory Circuit *via* a Synaptic Ampk-Dependent Positive Feedback Loop. Cell (2011) 146(6):992–1003. doi: 10.1016/j.cell.2011.07.039 21925320 PMC3209501

[144] Lu S Guan J-L Wang Q-P Uehara K Yamada S Goto N. Immunocytochemical Observation of Ghrelin-Containing Neurons in the Rat Arcuate Nucleus. Neurosci Lett (2002) 321:157–60. doi: 10.1016/S0304-3940(01)02544-7 11880196

[145] Guan XM Yu H Palyha OC McKee KK Feighner SD Sirinathsinghji DJ. Distribution of Mrna Encoding the Growth Hormone Secretagogue Receptor in Brain and Peripheral Tissues. Brain Res Mol Brain Res (1997) 48(1):23–9. doi: 10.1016/s0169-328x(97)00071-5 9379845

[146] Tucci SA Rogers EK Korbonits M Kirkham TC. The Cannabinoid Cb1 Receptor Antagonist Sr141716 Blocks the Orexigenic Effects of Intrahypothalamic Ghrelin. Br J Pharmacol (2004) 143:520–3. doi: 10.1038/sj.bjp.0705968 PMC1575431 15381634

[147] Soria-Gómez E Massa F Bellocchio L Rueda-Orozco PE Ciofi P Cota D. Cannabinoid Type-1 Receptors in the Paraventricular Nucleus of the Hypothalamus Inhibit Stimulated Food Intake. Neuroscience (2014) 263:46–53. doi: 10.1016/j.neuroscience.2014.01.005 24434770

[148] Kola B Hubina E Tucci SA Kirkham TC Garcia EA Mitchell SE. Cannabinoids and Ghrelin Have Both Central and Peripheral Metabolic and Cardiac Effects *via* Amp-Activated Protein Kinase. J Biol Chem (2005) 280:25196–201. doi: 10.1074/jbc.C500175200 15899896

[149] Kalafateli AL Vallöf D Jörnulf JW Heilig M Jerlhag E. A Cannabinoid Receptor Antagonist Attenuates Ghrelin-Induced Activation of the Mesolimbic Dopamine System in Mice. Physiol Behav (2018) 184:211–9. doi: 10.1016/j.physbeh.2017.12.005 29221808

[150] Ge X Yang H Bednarek MA Galon-Tilleman H Chen P Chen M. Leap2 Is an Endogenous Antagonist of the Ghrelin Receptor. Cell Metab (2018) 27(2):461–9.e6. doi: 10.1016/j.cmet.2017.10.016 29233536

[151] Hardie DG Scott JW Pan DA Hudson ER. Management of Cellular Energy by the Amp-Activated Protein Kinase System. FEBS Lett (2003) 546:113–20. doi: 10.1016/S0014-5793(03)00560-X 12829246

[152] Hardie DG Ross FA Hawley SA. Ampk: A Nutrient and Energy Sensor That Maintains Energy Homeostasis. Nat Rev (2012) 13:251–62. doi: 10.1038/nrm3311 PMC5726489 22436748

[153] Kola B Farkas I Christ-Crain M Wittmann G Lolli F Amin F. The Orexigenic Effect of Ghrelin Is Mediated Through Central Activation of the Endogenous Cannabinoid System. PloS One (2008) 3:e1797. doi: 10.371/journal.pone.0001797 18335063 PMC2258435

[154] Lim CT Kola B Feltrin D Perez-Tilve D Tschöp MH Grossman AB. Ghrelin and Cannabinoids Require the Ghrelin Receptor to Affect Cellular Energy Metabolism. Mol Cell Endocrinol (2013) 365:303–8. doi: 10.1016/j.mce.2012.11.007 PMC3566541 23178796

[155] Minokoshi Y Alquier T Furukawa N Kim Y-B Lee A Xue B. Amp-Kinase Regulates Food Intake by Responding to Hormonal and Nutrient Signals in the Hypothalamus. Nature (2004) 428:569–74. doi: 10.1038/nature02440 15058305

[156] Claret M Smith MA Batterham RL Selman C Choudhury AI Fryer LGD. Ampk Is Essential for Energy Homeostasis Regulation and Glucose Sensing by Pomc and Agrp Neurons. J Clin Invest (2007) 117:2325–36. doi: 10.1172/JCI31516 PMC1934578 17671657

[157] Dagon Y Hur E Zheng B Wellenstein K Cantley LC Kahn BB. P70s6 Kinase Phosphorylates Ampk on Serine 491 to Mediate Leptin's Effect on Food Intake. Cell Metab (2012) 16(1):104–12. doi: 10.1016/j.cmet.2012.05.010 PMC3407689 22727014

[158] Hawley SA Ross FA Gowans GJ Tibarewal P Leslie NR Hardie DG. Phosphorylation by Akt Within the St Loop of Ampk-A1 Down-Regulates its Activation in Tumour Cells. Biochem J (2014) 459(2):275–87. doi: 10.1042/bj20131344 PMC4052680 24467442

[159] Sohn JW. Ion Channels in the Central Regulation of Energy and Glucose Homeostasis. Front Neurosci (2013) 7:85. doi: 10.3389/fnins.2013.00085 23734095 PMC3661948

[160] Sun HS Feng ZP. Neuroprotective Role of Atp-Sensitive Potassium Channels in Cerebral Ischemia. Acta Pharmacol Sin (2013) 34(1):24–32. doi: 10.1038/aps.2012.138 23123646 PMC4086509

[161] Hille B. Potassium Channels and Chloride Channels. In: Hille B, editor. Ionic Channels of Excitable Membranes, 2 ed. Sunderland: Mass: Sinauer Associates, Inc (1992). p. 115–39.

[162] Hibino H Inanobe A Furutani K Murakami S Findlay I Kurachi Y. Inwardly Rectifying Potassium Channels: Their Structure, Function, and Physiological Roles. Physiol Rev (2010) 90:291–366. doi: 10.1152/physrev.00021.2009 20086079

[163] Inoue I Nagase H Kishi K Higuti T. Atp-Sensitive K+ Channel in the Mitochondrial Inner Membrane. Nature (1991) 352(6332):244–7. doi: 10.1038/352244a0 1857420

[164] Bajgar R Seetharaman S Kowaltowski AJ Garlid KD Paucek P. Identification and Properties of a Novel Intracellular (Mitochondrial) Atp-Sensitive Potassium Channel in Brain. J Biol Chem (2001) 276(36):33369–74. doi: 10.1074/jbc.M103320200 11441006

[165] Flagg TP Enkvetchakul D Koster JC Nichols CG. Muscle Katp Channels: Recent Insights to Energy Sensing and Myoprotection. Physiol Rev (2010) 90(3):799–829. doi: 10.1152/physrev.00027.2009 20664073 PMC3125986

[166] Thomzig A Laube G Prüss H Veh RW. Pore-Forming Subunits of K-Atp Channels, Kir6.1 and Kir6.2, Display Prominent Differences in Regional and Cellular Distribution in the Rat Brain. J Comp Neurol (2005) 484(3):313–30. doi: 10.1002/cne.20469 15739238

[167] DiLeone RJ Georgescu D Nestler EJ. Lateral Hypothalamic Neuropeptides in Reward and Drug Addiction. Life Sci (2003) 73:759–68. doi: 10.1016/S0024-3205(03)00408-9 12801597

[168] Georgescu D Sears RM Hommel JD Barrot M Bolaños CA Marsh DJ. The Hypothalamic Neuropeptide Melanin-Concentrating Hormone Acts in the Nucleus Accumbens to Modulate Feeding Behavior and Forced-Swim Performance. J Neurosci (2005) 25:2933–40. doi: 10.1523/JNEUROSCI.1714-04.2005 PMC6725126 15772353

[169] McClung CA Nestler EJ Zachariou V. Regulation of Gene Expression by Chronic Morphine and Morphine Withdrawal in the Locus Ceruleus and Ventral Tegmental Area. J Neurosci (2005) 25:6005–15. doi: 10.1523/JNEUROSCI.0062-05.2005 PMC6724795 15976090

[170] Carlsson A Falck B Hillarp NA. Cellular Localization of Brain Monoamines. Acta Physiol Scand Suppl (1962) 56(196):1–28. 14018711

[171] Dahlstroem A Fuxe K. Evidence for the Existence of Monoamine-Containing Neurons in the Central Nervous System. I. Demonstration of Monoamines in the Cell Bodies of Brain Stem Neurons. Acta Physiol Scand Suppl (1964) Suppl 232:1–55. 14229500

[172] Ehringer H Hornykiewicz O. Distribution of Noradrenaline and Dopamine (3-Hydroxytyramine) in the Human Brain and Their Behavior in Diseases of the Extrapyramidal System. Parkinsonism Relat Disord (1998) 4(2):53–7. doi: 10.1016/s1353-8020(98)00012-1 18591088

[173] Moore KE. Hypothalamic Dopaminergic Neuronal Systems. In: Meltzer HY, editor. Psychopharmacology: The Third Generation of Progress. New York: Raven Press (1987). p. 127–39.

[174] Beier KT Steinberg EE DeLoach KE Xie S Miyamichi K Schwarz L. Circuit Architecture of Vta Dopamine Neurons Revealed by Systematic Input-Output Mapping. Cell (2015) 162(3):622–34. doi: 10.1016/j.cell.2015.07.015 PMC4522312 26232228

[175] Tan KR Yvon C Turiault M Mirzabekov JJ Doehner J Labouebe G. Gaba Neurons of the Vta Drive Conditioned Place Aversion. Neuron (2012) 73:1173–83. doi: 10.1016/j.neuron.2012.02.015 PMC6690362 22445344

[176] van Zessen R Phillips JL Budygin EA Stuber GD. Activation of Vta Gaba Neurons Disrupts Reward Consumption. Neuron (2012) 73(6):1184–94. doi: 10.1016/j.neuron.2012.02.016 PMC3314244 22445345

[177] Jennings JH Sparta DR Stamatakis AM Ung RL Pleil KE Kash TL. Distinct Extended Amygdala Circuits for Divergent Motivational States. Nature (2013) 496:224–8. doi: 10.1038/nature12041 PMC3778934 23515155

[178] Tsai H-C Zhang F Adamantidis A Stuber GD Bonci A De Lecea L. Phasic Firing in Dopaminergic Neurons is Sufficient for Behavioral Conditioning. Science (2009) 324:1080–4. doi: 10.1126/science.1168878 PMC5262197 19389999

[179] Witten IB Steinberg EE Lee SY Davidson TJ Zalocusky KA Brodsky M. Recombinase-Driver Rat Lines: Tools, Techniques, and Optogenetic Application to Dopamine-Mediated Reinforcement. Neuron (2011) 72:721–33. doi: 10.1016/j.neuron.2011.10.028 PMC3282061 22153370

[180] Ilango A Kesner AJ Keller KL Stuber GD Bonci A Ikemoto S. Similar Roles of Substantia Nigra and Ventral Tegmental Dopamine Neurons in Reward and Aversion. J Neurosci (2014) 34:817–22. doi: 10.1523/JNEUROSCI.1703-13.2014 PMC3891961 24431440

[181] Steinberg EE Boivin JR Saunders BT Witten IB Deisseroth K Janak PH. Positive Reinforcement Mediated by Midbrain Dopamine Neurons Requires D1 and D2 Receptor Activation in the Nucleus Accumbens. PloS One (2014) 9(4):e94771. doi: 10.1371/journal.pone.0094771 24733061 PMC3986242

[182] Chuhma N Zhang H Masson J Zhuang X Sulzer D Hen R. Dopamine Neurons Mediate a Fast Excitatory Signal *via* Their Glutamatergic Synapses. J Neurosci (2004) 24(4):972–81. doi: 10.1523/jneurosci.4317-03.2004 PMC6729804 14749442

[183] Sulzer D Joyce MP Lin L Geldwert D Haber SN Hattori T. Dopamine Neurons Make Glutamatergic Synapses In Vitro. J Neurosci (1998) 18(12):4588–602. doi: 10.1523/jneurosci.18-12-04588.1998 PMC6792695 9614234

[184] Hnasko TS Chuhma N Zhang H Goh GY Sulzer D Palmiter RD. Vesicular Glutamate Transport Promotes Dopamine Storage and Glutamate Corelease *In Vivo* . Neuron (2010) 65(5):643–56. doi: 10.1016/j.neuron.2010.02.012 PMC2846457 20223200

[185] Fortin GM Bourque MJ Mendez JA Leo D Nordenankar K Birgner C. Glutamate Corelease Promotes Growth and Survival of Midbrain Dopamine Neurons. J Neurosci (2012) 32(48):17477–91. doi: 10.1523/jneurosci.1939-12.2012 PMC6621856 23197738

[186] Prado L Luis-Islas J Sandoval OI Puron L Gil MM Luna A. Activation of Glutamatergic Fibers in the Anterior Nac Shell Modulates Reward Activity in the Anacsh, the Lateral Hypothalamus, and Medial Prefrontal Cortex and Transiently Stops Feeding. J Neurosci (2016) 36(50):12511–29. doi: 10.1523/jneurosci.1605-16.2016 PMC6705665 27974611

[187] Qu N He Y Wang C Xu P Yang Y Cai X. A Pomc-Originated Circuit Regulates Stress-Induced Hypophagia, Depression, and Anhedonia. Mol Psychiatry (2020) 25:1006–21. doi: 10.1038/s41380-019-0506-1 PMC7056580 31485012

[188] Nieh EH Vander Weele CM Matthews GA Presbrey KN Wichmann R Leppla CA. Inhibitory Input From the Lateral Hypothalamus to the Ventral Tegmental Area Disinhibits Dopamine Neurons and Promotes Behavioral Activation. Neuron (2016) 90(6):1286–98. doi: 10.1016/j.neuron.2016.04.035 PMC4961212 27238864

[189] Nieh EH Matthews GA Allsop SA Presbrey KN Leppla CA Wichmann R. Decoding Neural Circuits That Control Compulsive Sucrose Seeking. Cell (2015) 160(3):528–41. doi: 10.1016/j.cell.2015.01.003 PMC4312417 25635460

[190] Borgland SL Taha SA Sarti F Fields HL Bonci A. Orexin a in the Vta Is Critical for the Induction of Synaptic Plasticity and Behavioral Sensitization to Cocaine. Neuron (2006) 49(4):589–601. doi: 10.1016/j.neuron.2006.01.016 16476667

[191] Zheng H Patterson LM Berthoud HR. Orexin Signaling in the Ventral Tegmental Area Is Required for High-Fat Appetite Induced by Opioid Stimulation of the Nucleus Accumbens. J Neurosci (2007) 27(41):11075–82. doi: 10.1523/jneurosci.3542-07.2007 PMC6672863 17928449

[192] Chung S Hopf FW Nagasaki H Li CY Belluzzi JD Bonci A. The Melanin-Concentrating Hormone System Modulates Cocaine Reward. Proc Natl Acad Sci USA (2009) 106(16):6772–7. doi: 10.1073/pnas.0811331106 PMC2672513 19342492

[193] Mul JD la Fleur SE Toonen PW Afrasiab-Middelman A Binnekade R Schetters D. Chronic Loss of Melanin-Concentrating Hormone Affects Motivational Aspects of Feeding in the Rat. PloS One (2011) 6(5):e19600. doi: 10.1371/journal.pone.0019600 21573180 PMC3088702

[194] de Vrind VAJ van 't Sant LJ Rozeboom A Luijendijk-Berg MCM Omrani A Adan RAH. Leptin Receptor Expressing Neurons in the Substantia Nigra Regulate Locomotion, and in the Ventral Tegmental Area Motivation and Feeding. Front Endocrinol (Lausanne) (2021) 12:680494. doi: 10.3389/fendo.2021.680494 34276560 PMC8281287

[195] Schiffino FL Siemian JN Petrella M Laing BT Sarsfield S Borja CB. Activation of a Lateral Hypothalamic-Ventral Tegmental Circuit Gates Motivation. PloS One (2019) 14(7):e0219522. doi: 10.1371/journal.pone.0219522 31291348 PMC6619795

[196] Siemian JN Arenivar MA Sarsfield S Borja CB Russell CN Aponte Y. Lateral Hypothalamic Lepr Neurons Drive Appetitive But Not Consummatory Behaviors. Cell Rep (2021) 36(8):109615. doi: 10.1016/j.celrep.2021.109615 34433027 PMC8423025

[197] Liu JJ Mukherjee D Haritan D Ignatowska-Jankowska B Liu J Citri A. High on Food: The Interaction Between the Neural Circuits for Feeding and for Reward. Front Biol (Beijing) (2015) 10(2):165–76. doi: 10.1007/s11515-015-1348-0 PMC5940344 29750082

[198] Volkow ND Wise RA Baler R. The Dopamine Motive System: Implications for Drug and Food Addiction. Nat Rev Neurosci (2017) 18:741–52. doi: 10.1038/nrn.2017.130 29142296

[199] Rada P Avena NM Hoebel BG. Daily Bingeing on Sugar Repeatedly Releases Dopamine in the Accumbens Shell. Neuroscience (2005) 134:737–44. doi: 10.1016/j.neuroscience.2005.04.043 15987666

[200] Avena NM Bocarsly ME. Dysregulation of Brain Reward Systems in Eating Disorders: Neurochemical Information From Animal Models of Binge Eating, Bulimia Nervosa, and Anorexia Nervosa. Neuropharmacology (2012) 63:87–96. doi: 10.1016/j.neuropharm.2011.11.010 22138162 PMC3366171

[201] Berridge KC. Food Reward: Brain Substrates of Wanting and Liking. Neurosci Biobehav Rev (1996) 20:1–25. doi: 10.1016/0149-7634(95)00033-B 8622814

[202] Berridge KC. 'Liking' and 'Wanting' Food Rewards: Brain Substrates and Roles in Eating Disorders. Physiol Behav (2009) 97:537–50. doi: 10.1016/j.physbeh.2009.02.044 PMC2717031 19336238

[203] Berridge KC. Measuring Hedonic Impact in Animals and Infants: Microstructure of Affective Taste Reactivity Patterns. Neurosci Biobehav Rev (2000) 24:173–98. doi: 10.1016/S0149-7634(99)00072-X 10714382

[204] Hommel JD Trinko R Sears RM Georgescu D Liu Z-W Gao X-B. Leptin Receptor Signaling in Midbrain Dopamine Neurons Regulates Feeding. Neuron (2006) 51:801–10. doi: 10.1016/j.neuron.2006.08.023 16982424

[205] Morton GJ Blevins JE Kim F Matsen M Figlewicz DP. The Action of Leptin in the Ventral Tegmental Area to Decrease Food Intake Is Dependent on Jak-2 Signaling. Am J Physiol Endocrinol Metab (2009) 297(1):E202–10. doi: 10.1152/ajpendo.90865.2008 PMC2711664 19435852

[206] Mebel DM Wong JC Dong YJ Borgland SL. Insulin in the Ventral Tegmental Area Reduces Hedonic Feeding and Suppresses Dopamine Concentration *via* Increased Reuptake. Eur J Neurosci (2012) 36(3):2336–46. doi: 10.1111/j.1460-9568.2012.08168.x PMC5239666 22712725

[207] Liu S Labouèbe G Karunakaran S Clee SM Borgland SL. Effect of Insulin on Excitatory Synaptic Transmission Onto Dopamine Neurons of the Ventral Tegmental Area in a Mouse Model of Hyperinsulinemia. Nutr Diabetes (2013) 3(12):e97. doi: 10.1038/nutd.2013.38 24336291 PMC3877429

[208] Liu S Globa AK Mills F Naef L Qiao M Bamji SX. Consumption of Palatable Food Primes Food Approach Behavior by Rapidly Increasing Synaptic Density in the Vta. Proc Natl Acad Sci USA (2016) 113(9):2520–5. doi: 10.1073/pnas.1515724113 PMC4780604 26884159

[209] Naleid AM Grace MK Cummings DE Levine AS. Ghrelin Induces Feeding in the Mesolimbic Reward Pathway Between the Ventral Tegmental Area and the Nucleus Accumbens. Peptides (2005) 26(11):2274–9. doi: 10.1016/j.peptides.2005.04.025 16137788

[210] Abizaid A Liu Z-W Andrews ZB Shanabrough M Borok E Elsworth JD. Ghrelin Modulates the Activity and Synaptic Input Organization of Midbrain Dopamine Neurons While Promoting Appetite. J Clin Invest (2006) 116:3229–39. doi: 10.1172/JCI29867 PMC1618869 17060947

[211] Valdivia S Cornejo MP Reynaldo M De Francesco PN Perello M. Escalation in High Fat Intake in a Binge Eating Model Differentially Engages Dopamine Neurons of the Ventral Tegmental Area and Requires Ghrelin Signaling. Psychoneuroendocrinology (2015) 60:206–16. doi: 10.1016/j.psyneuen.2015.06.018 26186250

[212] Gastelum C Perez L Hernandez J Le N Vahrson I Sayers S. Adaptive Changes in the Central Control of Energy Homeostasis Occur in Response to Variations in Energy Status. Int J Mol Sci (2021) 22(5):2728. doi: 10.3390/ijms22052728 33800452 PMC7962960

[213] Janssen I Powell LH Crawford S Lasley B Sutton-Tyrrell K. Menopause and the Metabolic Syndrome: The Study of Women's Health Across the Nation. Arch Intern Med (2008) 168(14):1568–75. doi: 10.1001/archinte.168.14.1568 PMC2894539 18663170

[214] Gustafsson PE Persson M Hammarström A. Life Course Origins of the Metabolic Syndrome in Middle-Aged Women and Men: The Role of Socioeconomic Status and Metabolic Risk Factors in Adolescence and Early Adulthood. Ann Epidemiol (2011) 21(2):103–10. doi: 10.1016/j.annepidem.2010.08.012 21184951

[215] Hoek HW. Incidence, Prevalence and Mortality of Anorexia Nervosa and Other Eating Disorders. Curr Opin Psychiatry (2006) 19:389–94. doi: 10.1097/01.yco.0000228759.95237.78 16721169

[216] Yang L Colditz GA. Prevalence of Overweight and Obesity in the United States 2007-2012. JAMA Intern Med (2015) 175:1412–3. doi: 10.1001/jamainternmed.2015.2405 PMC4625533 26098405

[217] Hetherington MM MacDiarmid JI. "Chocolate Addiction": A Preliminary Study of Its Description and its Relationship to Problem Eating. Appetite (1993) 21(3):233–46. doi: 10.1006/appe.1993.1042 8141595

[218] Tuomisto T Hetherington MM Morris MF Tuomisto MT Turjanmaa V Lappalainen R. Psychological and Physiological Characteristics of Sweet Food "Addiction". Int J Eat Disord (1999) 25(2):169–75. doi: 10.1002/(sici)1098-108x(199903)25:2<169::aid-eat6>3.0.co;2-b 10065394

[219] Lafay L Thomas F Mennen L Charles MA Eschwege E Borys JM. Gender Differences in the Relation Between Food Cravings and Mood in an Adult Community: Results From the Fleurbaix Laventie Ville Santé Study. Int J Eat Disord (2001) 29(2):195–204. doi: 10.1002/1098-108x(200103)29:2<195::aid-eat1009>3.0.co;2-n 11429982

[220] Zellner DA Garriga-Trillo A Rohm E Centeno S Parker S. Food Liking and Craving: A Cross-Cultural Approach. Appetite (1999) 33:61–70. doi: 10.1006/appe.1999.0234 10447980

[221] Frank S Laharnar N Kullmann S Veit R Canova C Hegner YL. Processing of Food Pictures: Influence of Hunger, Gender and Calorie Content. Brain Res (2010) 1350:159–66. doi: 10.1016/j.brainres.2010.04.030 20423700

[222] Weingarten HP Elston D. Food Cravings in a College Population. Appetite (1991) 17:167–75. doi: 10.1016/0195-6663(91)90019-O 1799279

[223] Wang GJ Volkow ND Telang F Jayne M Ma J Rao M. Exposure to Appetitive Food Stimuli Markedly Activates the Human Brain. Neuroimage (2004) 21(4):1790–7. doi: 10.1016/j.neuroimage.2003.11.026 15050599

[224] Uher R Treasure J Heining M Brammer MJ Campbell IC. Cerebral Processing of Food-Related Stimuli: Effects of Fasting and Gender. Behav Brain Res (2006) 169:111–9. doi: 10.1016/j.bbr.2005.12.008 16445991

[225] Wang GJ Volkow ND Telang F Jayne M Ma Y Pradhan K. Evidence of Gender Differences in the Ability to Inhibit Brain Activation Elicited by Food Stimulation. Proc Natl Acad Sci (2009) 106(4):1249–54. doi: 10.1073/pnas.0807423106 PMC2633545 19164587

[226] Imperatori C Innamorati M Tamburello S Continisio M Contardi A Tamburello A. Gender Differences in Food Craving Among Overweight and Obese Patients Attending Low Energy Diet Therapy: A Matched Case-Control Study. Eat Weight Disord (2013) 18(3):297–303. doi: 10.1007/s40519-013-0054-7 23904055

[227] Hallam J Boswell RG DeVito EE Kober H. Gender-Related Differences in Food Craving and Obesity. Yale J Biol Med (2016) 89:161–73. PMC4918881 27354843

[228] Oswald KD Murdaugh DL King VL Boggiano MM. Motivation for Palatable Food Despite Consequences in an Animal Model of Binge Eating. Int J Eat Disord (2011) 44(3):203–11. doi: 10.1002/eat.20808 PMC2941549 20186718

[229] Yu Z Indelicato NA Fuglestad P Tan M Bane L. Sex Differences in Disordered Eating and Food Addiction Among College Students. Appetite (2018) 129:12–8. doi: 10.1016/j.appet.2018.06.028 29935291

[230] Asarian L Geary N. Cyclic Estradiol Treatment Normalizes Body Weight and Restores Physiologic Patterns of Spontaneous Feeding and Sexual Receptivity in Ovariectomized Rats. Horm Behav (2002) 42:461–71. doi: 10.1006/hbeh.2002.1835 12488112

[231] Asarian L Geary N. Modulation of Appetite by Gonadal Steroid Hormones. Phil Trans R Soc B (2006) 361:1251–63. doi: 10.1098/rstb.2006.1860 PMC1642706 16815802

[232] Gao Q Mezei G Nie Y Rao Y Choi CS Bechmann I. Anorectic Estrogen Mimics Leptin's Effect on the Rewiring of Melanocortin Cells and Stat3 Signaling in Obese Animals. Nat Med (2007) 13:89–94. doi: 10.1038/nm1525 17195839

[233] Santollo J Wiley MD Eckel LA. Acute Activation of Erα Decreases Food Intake, Meal Size and Body Weight in Ovariectomized Rats. Am J Physiol Regul Integr Comp Physiol (2007) 293:R2194–R201. doi: 10.1152/ajpregu.00385.2007 17942491

[234] Kellert BA Nguyen MC Nguyen C Nguyen QH Wagner EJ. Estrogen Rapidly Attenuates Cannabinoid-Induced Changes in Energy Homeostasis. Eur J Pharmacol (2009) 622:15–24. doi: 10.1016/j.ejphar.2009.09.001 19758570 PMC2790528

[235] Roepke TA Bosch MA Rick EA Lee B Wagner EJ Seidlova-Wuttke D. Contribution of a Membrane Estrogen Receptor to the Estrogenic Regulation of Body Temperature and Energy Homeostasis. Endocrinology (2010) 151:4926–37. doi: 10.1210/en.2010-0573 PMC2946146 20685867

[236] Dellovade TL Merchenthaler I. Estrogen Regulation of Neurokinin B Gene Expression in the Mouse Arcuate Nucleus is Mediated by Estrogen Receptor Alpha. Endocrinology (2004) 145(2):736–42. doi: 10.1210/en.2003-0894 14592957

[237] Heine PA Taylor JA Iwamoto GA Lubahn DB Cooke PS. Increased Adipose Tissue in Male and Female Estrogen Receptor-Alpha Knockout Mice. Proc Natl Acad Sci USA (2000) 97(23):12729–34. doi: 10.1073/pnas.97.23.12729 PMC18832 11070086

[238] Qiu J Rønnekleiv OK Kelly MJ. Modulation of Hypothalamic Neuronal Activity Through a Novel G-Protein-Coupled Estrogen Receptor. Steroids (2008) 73:985–91. doi: 10.1016/j.steroids.2007.11.008 PMC5466077 18342349

[239] Hu P Liu J Yasrebi A Gotthardt JD Bello NT Pang ZP. Gq Protein-Coupled Membrane-Initiated Estrogen Signaling Rapidly Excites Corticotropin-Releasing Hormone Neurons in the Hypothalamic Paraventricular Nucleus in Female Mice. Endocr (2016) 157:3604–20. doi: 10.1210/en.2016-1191 PMC5007888 27387482

[240] Conde K Meza C Kelly MJ Sinchak K Wagner EJ. Estradiol Rapidly Attenuates Orl-1 Receptor-Mediated Inhibition of Proopiomelanocortin Neurons *via* G_q_-Coupled, Membrane-Initiated Signaling. Neuroendocrinology (2016) 103:787–805. doi: 10.1159/000443765 26765570 PMC4947458

[241] Lagrange AH Ronnekleiv OK Kelly MJ. The Potency of μ-Opioid Hyperpolarization of Hypothalamic Arcuate Neurons Is Rapidly Attenuated by 17β-Estradiol. J Neurosci (1994) 14:6196–204. doi: 10.1523/JNEUROSCI.14-10-06196.1994 PMC6576966 7931572

[242] Qiu J Bosch MA Tobias SC Grandy DK Scanlan TS Rønnekleiv OK. Rapid Signaling of Estrogen in Hypothalamic Neurons Involves a Novel G-Protein-Coupled Estrogen Receptor That Activates Protein Kinase C. J Neurosci (2003) 23:9529–40. doi: 10.1523/JNEUROSCI.23-29-09529.2003 PMC6740471 14573532

[243] Washburn N Borgquist A Wang K Jeffery GS Kelly MJ Wagner EJ. Receptor Subtypes and Signal Transduction Mechanisms Contributing to the Estrogenic Attenuation of Cannabinoid-Induced Changes in Energy Homeostasis. Neuroendocrinology (2013) 97:160–75. doi: 10.1159/000338669 PMC3702272 22538462

[244] Smith AW Bosch MA Wagner EJ Rønnekleiv OK Kelly MJ. The Membrane Estrogen Receptor Ligand Stx Rapidly Enhances Gabaergic Signaling in Npy/Agrp Neurons: Role in Mediating the Anorexigenic Effects of 17β-Estradiol. Am J Physiol Endocrinol Metab (2013) 305:E362–640. doi: 10.1152/ajpendo.00281.2013 PMC3761166 23820624

[245] Romero-Picó A Novelle MG Al-Massadi O Beiroa D Tojo M Heras V. Kappa-Opioid Receptor Blockade Ameliorates Obesity Caused by Estrogen Withdrawal *via* Promotion of Energy Expenditure Through Mtor Pathway. Int J Mol Sci (2022) 23(6):3118. doi: 10.3390/ijms23063118 35328539 PMC8953356

[246] Xu Y Nedungadi TP Zhu L Sobhani N Irani BG Davis KE. Distinct Hypothalamic Neurons Mediate Estrogenic Effects on Energy Homeostasis and Reproduction. Cell Metab (2011) 14:453–65. doi: 10.1016/j.cmet.2011.08.009 PMC3235745 21982706

[247] Dhillon SS Belsham DD. Estrogen Inhibits Npy Secretion Through Membrane-Associated Estrogen Receptor (Er)-α in Clonal, Immortalized Hypothalamic Neurons. Int J Obes (2011) 35:198–207. doi: 10.1038/ijo.2010.124 20548307

[248] Musatov S Chen W Pfaff DW Mobbs CV Yang X-J Clegg DJ. Silencing of Estrogen Receptor α in the Ventromedial Nucleus of the Hypothalamus Leads to Metabolic Syndrome. Proc Natl Acad Sci (2007) 104:2501–6. doi: 10.1073/pnas.0610787104 PMC1892990 17284595

[249] Martínez de Morentin PB González-García I Martins L Lage R Fernández-Mallo D Martínez-Sánchez N. Estradiol Regulates Brown Adipose Tissue Thermogenesis *via* Hypothalamic Ampk. Cell Metab (2014) 20(1):41–53. doi: 10.1016/j.cmet.2014.03.031 24856932 PMC4082097

[250] Olofsson LE Pierce AA Xu AW. Functional Requirement of Agrp and Npy Neurons in Ovarian Cycle-Dependent Regulation of Food Intake. Proc Natl Acad Sci (2009) 106:15932–7. doi: 10.1073/pnas.0904747106 PMC2747221 19805233

[251] Clegg DJ Brown LM Zigman JM Kemp CJ Strader AD Benoit SC. Estradiol-Dependent Decrease in the Orexigenic Potency of Ghrelin in Female Rats. Diabetes (2007) 56:1051–8. doi: 10.2337/db06-0015 17251274

[252] Roepke TA Qiu J Smith AW Rønnekleiv OK Kelly MJ. Fasting and 17β-Estradiol Differentially Modulate the M-Current in Neuropeptide Y Neurons. J Neurosci (2011) 31:11825–35. doi: 10.1523/JNEUROSCI.1395-11.2011 PMC3243733 21849543

[253] Stincic TL Bosch MA Hunker AC Juarez B Connors AM Zweifel LS. Crispr Knockdown of Kcnq3 Attenuates the M-Current and Increases Excitability of Npy/Agrp Neurons to Alter Energy Balance. Mol Metab (2021) 49:101218. doi: 10.1016/j.molmet.2021.101218 33766732 PMC8093934

[254] Roepke TA Smith AW Rønnekleiv OK Kelly MJ. Serotonin 5-Ht2c Receptor-Mediated Inhibition of the M-Current in Hypothalamic Pomc Neurons. Am J Physiol Endocrinol Metab (2012) 302(11):E1399–406. doi: 10.1152/ajpendo.00565.2011 PMC3378066 22436698

[255] Lee DK Jeong JH Oh S Jo YH. Apelin-13 Enhances Arcuate Pomc Neuron Activity *via* Inhibiting M-Current. PloS One (2015) 10(3):e0119457. doi: 10.1371/journal.pone.0119457 25782002 PMC4363569

[256] Fu LY van den Pol AN. Kisspeptin Directly Excites Anorexigenic Proopiomelanocortin Neurons But Inhibits Orexigenic Neuropeptide Y Cells by an Indirect Synaptic Mechanism. J Neurosci (2010) 30(30):10205–19. doi: 10.1523/jneurosci.2098-10.2010 PMC2933146 20668204

[257] Nestor CC Qiu J Padilla SL Zhang C Bosch MA Fan W. Optogenetic Stimulation of Arcuate Nucleus Kiss1 Neurons Reveals a Steroid-Dependent Glutamatergic Input to Pomc and Agrp Neurons in Male Mice. Mol Endocrinol (2016) 30(6):630–44. doi: 10.1210/me.2016-1026 PMC4884339 27093227

[258] Conde K Roepke TA. 17β-Estradiol Increases Arcuate Kndy Neuronal Sensitivity to Ghrelin Inhibition of the M-Current in Female Mice. Neuroendocrinology (2020) 110(7-8):582–94. doi: 10.1159/000503146 PMC7056582 31484184

[259] Qiu J Rivera HM Bosch MA Padilla SL Stincic TL Palmiter RD. Estrogenic-Dependent Glutamatergic Neurotransmission From Kisspeptin Neurons Governs Feeding Circuits in Females. eLife (2018) 7:e35656. doi: 10.7554/eLife.35656 30079889 PMC6103748

[260] Richard JE Lopez-Ferreras L Anderberg RH Olandersson K Skibicka KP. Estradiol Is a Critical Regulator of Food-Reward Behavior. Psychoneuroendocrinology (2017) 78:193–202. doi: 10.1016/j.psyneuen.2017.01.014 28214679

[261] Borgquist A Meza C Wagner EJ. The Role of Amp-Activated Protein Kinase in the Androgenic Potentiation of Cannabinoid-Induced Changes in Energy Homeostasis. Am J Physiol Endocrinol Metab (2015) 308:E482–E95. doi: 10.1152/ajpendo.00421.2014 PMC4360013 25550281

[262] Conde K Fabelo C Krause WC Propst R Goethel J Fischer D. Testosterone Rapidly Augments Retrograde Endocannabinoid Signaling in Proopiomelanocortin Neurons to Suppress Glutamatergic Input From Steroidogenic Factor 1 Neurons *via* Upregulation of Diacylglycerol Lipase-α. Neuroendocrinology (2017) 105:341–56. doi: 10.1159/000453370 PMC5839320 27871072

[263] Hernandez J Fabelo C Perez L Moore C Chang R Wagner EJ. Nociceptin/Orphanin Fq Modulates Energy Homeostasis Through Inhibition of Neurotransmission at Vmn Sf-1/Arc Pomc Synapses in a Sex- and Diet-Dependent Manner. Biol Sex Diff (2019) 10(1):9. doi: 10.1186/s13293-019-0220-3 PMC6373052 30755252

[264] Miyata A Arimura A Dahl RR Minamino N Uehara A Jiang L. Isolation of a Novel 38 Residue-Hypothalamic Polypeptide Which Stimulates Adenylate Cyclase in Pituitary Cells. Biochem Biophys Res Commun (1989) 164(1):567–74. doi: 10.1016/0006-291x(89)91757-9 2803320

[265] Adams BA Lescheid DW Vickers ED Crim LW Sherwood NM. Pituitary Adenylate Cyclase-Activating Polypeptide and Growth Hormone-Releasing Hormone-Like Peptide in Sturgeon, Whitefish, Grayling, Flounder and Halibut: Cdna Sequence, Exon Skipping and Evolution. Regul Pept (2002) 109(1-3):27–37. doi: 10.1016/s0167-0115(02)00167-2 12409211

[266] Rudecki AP Gray SL. Pacap in the Defense of Energy Homeostasis. Trends Endocrinol Metab (2016) 27:620–32. doi: 10.1016/j.tem.2016.04.008 27166671

[267] Hirabayashi T Nakamachi T Shioda S. Discovery of Pacap and Its Receptors in the Brain. J Headache Pain (2018) 19(1):28. doi: 10.1186/s10194-018-0855-1 29619773 PMC5884755

[268] Vaudry D Gonzalez BJ Basille M Yon L Fournier A Vaudry H. Pituitary Adenylate Cyclase-Activating Polypeptide and its Receptors: From Structure to Functions. Pharmacol Rev (2000) 52(2):269–324. 10835102

[269] Filipsson K Kvist-Reimer M Ahrén B. The Neuropeptide Pituitary Adenylate Cyclase-Activating Polypeptide and Islet Function. Diabetes (2001) 50(9):1959–69. doi: 10.2337/diabetes.50.9.1959 11522660

[270] Arimura A Somogyvari-Vigh A Miyata A Mizuno K Coy DH Kitada C. Tissue Distribution of Pacap as Determined by Ria: Highly Abundant in the Rat Brain and Testes. Endocrinology (1991) 129:2787–9. doi: 10.1210/endo-129-5-2787 1935809

[271] Mounien L Do Rego J-C Bizet P Boutelet I Gourcerol G Fournier A. Pituitary Adenylate Cyclase-Activating Polypeptide Inhibits Food Intake in Mice Through Activation of the Hypothalamic Melanocortin System. Neuropsychopharmacol (2009) 34:424–35. doi: 10.1038/npp.2008.73 18536705

[272] Resch JM Maunze B Gerhardt AK Magnuson SK Phillips KA Choi S. Intrahypothalamic Pituitary Adenylate Cyclase-Activating Polypeptide Regulates Energy Balance *via* Site-Specific Actions on Feeding and Metabolism. Am J Physiol Endocrinol Metab (2013) 305:E1452–E63. doi: 10.1152/ajpendo.00293.2013 PMC3882380 24148346

[273] Lutz-Bucher B Monnier D Koch B. Evidence for the Presence of Receptors for Pituitary Adenylate Cyclase-Activating Polypeptide in the Neurohypophysis That are Positively Coupled to Cyclic Amp Formation and Neurohypophyseal Hormone Secretion. Neuroendocrinology (1996) 64(2):153–61. doi: 10.1159/000127113 8857610

[274] Uchimura D Katafuchi T Hori T Yanaihara N. Facilitatory Effects of Pituitary Adenylate Cyclase Activating Polypeptide (Pacap) on Neurons in the Magnocellular Portion of the Rat Hypothalamic Paraventricular Nucleus (Pvn) *In Vitro* . J Neuroendocrinol (1996) 8:137–43. doi: 10.1111/j.1365-2826.1996.tb00834.x 8868261

[275] Shibuya I Kabashima N Tanaka K Setiadji VS Noguchi J Harayama N. Patch-Clamp Analysis of the Mechanism of Pacap-Induced Excitation in Rat Supraoptic Neurones. J Neuroendocrinol (1998) 10:759–68. doi: 10.1046/j.1365-2826.1998.00260.x 9792327

[276] Masuo Y Matsumoto Y Tokito F Tsuda M Fujino M. Effects of Vasoactive Intestinal Polypeptide (Vip) and Pituitary Adenylate Cyclase Activating Polypeptide (Pacap) on the Spontaneous Release of Acetylcholine From the Rat Hippocampus by Brain Microdialysis. Brain Res (1993) 611(2):207–15. doi: 10.1016/0006-8993(93)90504-g 8334515

[277] Wang G Pan J Tan Y-Y Sun X-K Zhang Y-F Zhou H-Y. Neuroprotective Effects of Pacap27 in Mice Model of Parkinson's Disease Involved in the Modulation of K(Atp) Subunits and D2 Receptors in the Striatum. Neuropeptides (2008) 42:267–76. doi: 10.1016/j.npep.2008.03.002 18440632

[278] Maasz G Zrinyi Z Reglodi D Petrovics D Rivnyak A Kiss T. Pituitary Adenylate Cyclase-Activating Polypeptide (Pacap) Has a Neuroprotective Function in Dopamine-Based Neurodegeneration in Rat and Snail Parkinsonian Models. Dis Model Mech (2017) 10(2):127–39. doi: 10.1242/dmm.027185 PMC5312006 28067625

[279] Gottschall PE Tatsuno I Miyata A Arimura A. Characterization and Distribution of Binding Sites for the Hypothalamic Peptide, Pituitary Adenylate Cyclase-Activating Polypeptide. Endocrinology (1990) 127:272–7. doi: 10.1210/endo-127-1-272 2361473

[280] Segal JP Stallings NR Lee CE Zhao L Socci N Viale A. Use of Laser-Capture Microdissection for the Identification of Marker Genes for the Ventromedial Hypothalamic Nucleus. J Neurosci (2005) 25:4181–8. doi: 10.1523/JNEUROSCI.0158-05.2005 PMC6724958 15843621

[281] Hawke Z Ivanov TR Bechtold DA Dhillon H Lowell BB Luckman SM. Pacap Neurons in the Hypothalamic Ventromedial Nucleus Are Targets of Central Leptin Signaling. J Neurosci (2009) 29:14828–35. doi: 10.1523/JNEUROSCI.1526-09.2009 PMC6666015 19940178

[282] Matsuda K Maruyama K Nakamachi T Miura T Uchiyama M Shioda S. Inhibitory Effects of Pituitary Adenylate Cyclase-Activating Polypeptide (Pacap) and Vasoactive Intestinal Peptide (Vip) on Food Intake in the Goldfish, Carassius Auratus. Peptides (2005) 26(9):1611–6. doi: 10.1016/j.peptides.2005.02.022 16112400

[283] Vu JP Goyal D Luong L Oh S Sanghu R Norris J. Pacap Intraperitoneal Treatment Suppresses Appetite and Food Intake *via* Pac1 Receptor in Mice by Inhibiting Ghrelin and Increasing Glp-1 and Leptin. Am J Physiol Gastrointest Liver Physiol (2015) 309:G816–G25. doi: 10.1152/ajpgi.00190.2015 PMC4652141 26336928

[284] Morley JE Horowitz M Morley PM Flood JF. Pituitary Adenylate Cyclase Activating Polypeptide (Pacap) Reduces Food Intake in Mice. Peptides (1992) 13(6):1133–5. doi: 10.1016/0196-9781(92)90019-y 1494495

[285] Tachibana T Tomonaga S Oikawa D Saito S Takagi T Saito ES. Pituitary Adenylate Cyclase Activating Polypeptide and Vasoactive Intestinal Peptide Inhibit Feeding in the Chick Brain by Different Mechanisms. Neurosci Lett (2003) 348(1):25–8. doi: 10.1016/s0304-3940(03)00646-3 12893417

[286] Resch JM Boisvert JP Hourigan AE Muller CR Yi SS Choi S. Stimulation of the Hypothalamic Ventromedial Nuclei by Pituitary Adenylate Cyclase-Activating Polypeptide Induces Hypophagia and Thermogenesis. Am J Physiol Regul Integr Comp Physiol (2011) 301:R1625–R34. doi: 10.1152/ajpregu.00334.2011 PMC3233848 21957159

[287] Hurley MM Maunze B Block ME Frenkel MM Reily MJ Kim E. Pituitary Adenylate-Cyclase Activating Polypeptide Regulates Hunger- and Palatability-Induced Binge Eating. Front Neurosci (2016) 10:383. doi: 10.3389/fnins.2016.00383 27597817 PMC4993128

[288] Le N Hernandez J Gastelum C Perez L Vahrson I Sayers S. Pituitary Adenylate Cyclase Activating Polypeptide Inhibits a(10) Dopamine Neurons and Suppresses the Binge-Like Consumption of Palatable Food. Neuroscience (2021) 478:49–64. doi: 10.1016/j.neuroscience.2021.09.016 34597709 PMC8608708

[289] Marquez P Bebawy D Lelièvre V Coûté AC Evans CJ Waschek JA. The Role of Endogenous Pacap in Motor Stimulation and Conditioned Place Preference Induced by Morphine in Mice. Psychopharmacol (Berl) (2009) 204(3):457–63. doi: 10.1007/s00213-009-1476-9 PMC4429892 19199096

[290] McMillan TR Forster MAM Short LI Rudecki AP Cline DL Gray SL. Melanotan Ii, a Melanocortin Agonist, Partially Rescues the Impaired Thermogenic Capacity of Pituitary Adenylate Cyclase-Activating Polypeptide Deficient Mice. Exp Physiol (2021) 106(2):427–37. doi: 10.1113/ep088838 33332767

[291] Jozsa R Nemeth J Tamas A Hollosy T Lubics A Jakab B. Short-Term Fasting Differentially Alters Pacap and Vip Levels in the Brains of Rat and Chicken. Ann N Y Acad Sci (2006) 1070:354–8. doi: 10.1196/annals.1317.044 16888191

[292] Kiss P Reglodi D Tamás A Lubics A Lengvári I Józsa R. Changes of Pacap Levels in the Brain Show Gender Differences Following Short-Term Water and Food Deprivation. Gen Comp Endocrinol (2007) 152(2-3):225–30. doi: 10.1016/j.ygcen.2006.12.012 17286974

[293] Nakata M Kohno D Shintani N Nemoto Y Hashimoto H Baba A. Pacap Deficient Mice Display Reduced Carbohydrate Intake and Pacap Activates Npy-Containing Neurons in the Rat Hypothalamic Arcuate Nucleus. Neurosci Lett (2004) 370(2-3):252–6. doi: 10.1016/j.neulet.2004.08.034 15488333

[294] Mounien L Bizet P Boutelet I Gourcerol G Basille M Gonzalez B. Expression of Pacap Receptor Mrnas by Neuropeptide Y Neurons in the Rat Arcuate Nucleus. Ann N Y Acad Sci (2006) 1070:457–61. doi: 10.1196/annals.1317.061 16888209

[295] Krashes MJ Shah BP Madara JC Olson DP Strochlic DE Garfield AS. An Excitatory Paraventricular Nucleus to Agrp Neuron Circuit That Drives Hunger. Nature (2014) 507:238–42. doi: 10.1038/nature12956 PMC3955843 24487620

[296] Nguyen TT Kambe Y Kurihara T Nakamachi T Shintani N Hashimoto H. Pituitary Adenylate Cyclase-Activating Polypeptide in the Ventromedial Hypothalamus Is Responsible for Food Intake Behavior by Modulating the Expression of Agouti-Related Peptide in Mice. Mol Neurobiol (2020) 57(4):2101–14. doi: 10.1007/s12035-019-01864-7 31927724

[297] Hurley MM Anderson EM Chen C Maunze B Hess EM Block ME. Acute Blockade of Pacap-Dependent Activity in the Ventromedial Nucleus of the Hypothalamus Disrupts Leptin-Induced Behavioral and Molecular Changes in Rats. Neuroendocrinology (2020) 110(3-4):271–81. doi: 10.1159/000501337 PMC6895395 31167202

[298] Choi DC Evanson NK Furay AR Ulrich-Lai YM Ostrander MM Herman JP. The Anteroventral Bed Nucleus of the Stria Terminalis Differentially Regulates Hypothalamic-Pituitary-Adrenocortical Axis Responses to Acute and Chronic Stress. Endocrinology (2008) 149(2):818–26. doi: 10.1210/en.2007-0883 PMC2219312 18039788

[299] Roman CW Lezak KR Kocho-Schellenberg M Garret MA Braas K May V. Excitotoxic Lesions of the Bed Nucleus of the Stria Terminalis (Bnst) Attenuate the Effects of Repeated Stress on Weight Gain: Evidence for the Recruitment of Bnst Activity by Repeated, But Not Acute, Stress. Behav Brain Res (2012) 227(1):300–4. doi: 10.1016/j.bbr.2011.11.010 PMC3242928 22101300

[300] Kocho-Schellenberg M Lezak KR Harris OM Roelke E Gick N Choi I. Pacap in the Bnst Produces Anorexia and Weight Loss in Male and Female Rats. Neuropsychopharmacology (2014) 39(7):1614–23. doi: 10.1038/npp.2014.8 PMC4023158 24434744

[301] Fink G. In Retrospect: Eighty Years of Stress. Nature (2016) 539(7628):175–6. doi: 10.1038/nature20473 27783596

[302] Schommer NC Hellhammer DH Kirschbaum C. Dissociation Between Reactivity of the Hypothalamus-Pituitary-Adrenal Axis and the Sympathetic-Adrenal-Medullary System to Repeated Psychosocial Stress. Psychosom Med (2003) 65(3):450–60. doi: 10.1097/01.psy.0000035721.12441.17 12764219

[303] Dallman MF Pecoraro NC la Fleur SE. Chronic Stress and Comfort Foods: Self-Medication and Abdominal Obesity. Brain Behav Immun (2005) 19(4):275–80. doi: 10.1016/j.bbi.2004.11.004 15944067

[304] Torres SJ Nowson CA. Relationship Between Stress, Eating Behavior, and Obesity. Nutrition (2007) 23(11-12):887–94. doi: 10.1016/j.nut.2007.08.008 17869482

[305] Warne JP. Shaping the Stress Response: Interplay of Palatable Food Choices, Glucocorticoids, Insulin and Abdominal Obesity. Mol Cell Endocrinol (2009) 300(1-2):137–46. doi: 10.1016/j.mce.2008.09.036 18984030

[306] Block JP He Y Zaslavsky AM Ding L Ayanian JZ. Psychosocial Stress and Change in Weight Among Us Adults. Am J Epidemiol (2009) 170(2):181–92. doi: 10.1093/aje/kwp104 PMC2727271 19465744

[307] Jastreboff AM Sinha R Lacadie C Small DM Sherwin RS Potenza MN. Neural Correlates of Stress- and Food Cue-Induced Food Craving in Obesity: Association With Insulin Levels. Diabetes Care (2013) 36(2):394–402. doi: 10.2337/dc12-1112 23069840 PMC3554293

[308] Rutters F Lemmens SG Born JM Bouwman F Nieuwenhuizen AG Mariman E. Genetic Associations With Acute Stress-Related Changes in Eating in the Absence of Hunger. Patient Educ Couns (2010) 79(3):367–71. doi: 10.1016/j.pec.2010.03.013 20409671

[309] Wang GJ Geliebter A Volkow ND Telang FW Logan J Jayne MC. Enhanced Striatal Dopamine Release During Food Stimulation in Binge Eating Disorder. Obes (Silver Spring) (2011) 19(8):1601–8. doi: 10.1038/oby.2011.27 PMC3144277 21350434

[310] Gearhardt AN Yokum S Orr PT Stice E Corbin WR Brownell KD. Neural Correlates of Food Addiction. Arch Gen Psychiatry (2011) 68(8):808–16. doi: 10.1001/archgenpsychiatry.2011.32 PMC3980851 21464344

[311] Sinha R. Chronic Stress, Drug Use, and Vulnerability to Addiction. Ann N Y Acad Sci (2008) 1141:105–30. doi: 10.1196/annals.1441.030 PMC2732004 18991954

[312] Fairburn CG Cooper Z Doll HA Norman P O'Connor M. The Natural Course of Bulimia Nervosa and Binge Eating Disorder in Young Women. Arch Gen Psychiatry (2000) 57(7):659–65. doi: 10.1001/archpsyc.57.7.659 10891036

[313] Bello NT Hajnal A. Dopamine and Binge Eating Behaviors. Pharmacol Biochem Behav (2010) 97(1):25–33. doi: 10.1016/j.pbb.2010.04.016 20417658 PMC2977997

[314] Hurley MM Robble MR Callan G Choi S Wheeler RA. Pituitary Adenylate Cyclase-Activiating Polypeptide (Pacap) Acts in the Nucleus Accumbens to Reduce Hedonic Drive. Int J Obes (2019) 43:928–32. doi: 10.1038/s41366-018-0154-6 PMC6363914 30082747

